# The CAPE1 peptide confers resistance against bacterial wilt in tomato

**DOI:** 10.1093/jxb/eraf145

**Published:** 2025-04-03

**Authors:** Weiqi Zhang, Marc Planas-Marquès, Moyan Liang, Qingshan Zhang, Annemarie Vermeulen, Farnusch Kaschani, Markus Kaiser, Frank L W Takken, Nuria S Coll, Marc Valls

**Affiliations:** Centre for Research in Agricultural Genomics (CRAG), CSIC-IRTA-UAB-UB, Campus UAB, 08193 Bellaterra, Spain; Centre for Research in Agricultural Genomics (CRAG), CSIC-IRTA-UAB-UB, Campus UAB, 08193 Bellaterra, Spain; Department of Genetics, Microbiology and Statistics, Universitat de Barcelona, 08028 Barcelona, Spain; Centre for Research in Agricultural Genomics (CRAG), CSIC-IRTA-UAB-UB, Campus UAB, 08193 Bellaterra, Spain; Department of Genetics, Microbiology and Statistics, Universitat de Barcelona, 08028 Barcelona, Spain; Centre for Research in Agricultural Genomics (CRAG), CSIC-IRTA-UAB-UB, Campus UAB, 08193 Bellaterra, Spain; Molecular Plant Pathology, Faculty of Science, Swammerdam Institute for Life Sciences, University of Amsterdam, Amsterdam, Netherlands; Analytics Core Facility Essen (ACE), Chemical Biology, Faculty of Biology, Universität Duisburg-Essen, ZMB, 45117 Essen, Germany; Chemical Biology, Faculty of Biology, Universität Duisburg-Essen, ZMB, 45117 Essen, Germany; Molecular Plant Pathology, Faculty of Science, Swammerdam Institute for Life Sciences, University of Amsterdam, Amsterdam, Netherlands; Centre for Research in Agricultural Genomics (CRAG), CSIC-IRTA-UAB-UB, Campus UAB, 08193 Bellaterra, Spain; Consejo Superior de Investigaciones Científicas (CSIC), 08001 Barcelona, Spain; Centre for Research in Agricultural Genomics (CRAG), CSIC-IRTA-UAB-UB, Campus UAB, 08193 Bellaterra, Spain; Department of Genetics, Microbiology and Statistics, Universitat de Barcelona, 08028 Barcelona, Spain; University of Ghent, Belgium

**Keywords:** Bacterial wilt, PR1 proteins, *Ralstonia solanacearum*, *Solanum lycopersicum*, tomato disease resistance, xylem proteome

## Abstract

Bacterial wilt caused by *Ralstonia solanacearum* is one of the most destructive bacterial diseases for which no effective treatment exists. There is an urgent need to understand the basis of resistance against this pathogen in order to engineer efficient strategies in the field. We previously demonstrated that resistant tomato plants limit bacterial movement in the apoplast and the xylem. As a first step to dissect the underlying mechanisms, we analysed the apoplast proteome upon challenge with *R. solanacearum* in the susceptible tomato cultivar Marmande and the resistant cultivar Hawaii 7996. Here, we described the xylem proteome in these same cultivars and compared it with the apoplastic proteome, revealing variety-dependent and infection-dependent changes. This proteomic analysis led to the identification of pathogenesis-related 1 (PR1) proteins as highly induced upon infection. Since PR1b was the most abundant PR1 protein in both the apoplast and the xylem, we concentrated on this family member to study the role of PR1s in the interaction between tomato and *R. solanacearum*. Surprisingly, lack of PR1b resulted in enhanced resistance to *R. solanacearum* in tomato, which could be due to an up-regulation of homologous genes in a compensatory effect as has been reported before. PR1 processing by an unknown protease in tomato results in the generation of the CAPE peptide. Treatment of tomato plants with the CAPE1 peptide resulted in restriction of *R. solanacearum* growth, via defence gene reprogramming. Future work in the lab will help determine which tomato secreted proteases cleave PR1s to generate CAPEs.

## Teaser

Resistance against bacterial wilt in tomato is linked to variety-specific proteomic changes; the CAPE1 peptide restricts *Ralstonia solancearum* growth *in planta* .

## Introduction

Bacterial wilt caused by *Ralstonia solanacearum* (some strains are also called *R. pseudosolanacearum*) is one of the most destructive bacterial diseases and affects over 200 plant species, including important crops such as tomato, potato, and peanut (Mansfield *et al.*, 2012). *Ralstonia solanacearum* is soil-borne and infects plants through the roots, migrating intercellularly through the apoplast until it reaches the xylem vessels, where it multiplies extensively and clogs infected vessels causing plant wilting (Vasse, 1995). Management of bacterial wilt remains difficult due to the aggressiveness of the pathogen and its long persistence in the environment (Mansfield *et al.*, 2012), with yield losses reaching up to 90% in some cases. In tomato, the cultivar Hawaii 7996 (H7996) is the most effective source of resistance against different *R. solanacearum* strains (Grimault *et al.*, 1994*a*; Prior *et al.*, 1996; Wang *et al.*, 1998).

Infection of the xylem vessels causes substantial metabolic changes in the xylem content and the surrounding parenchymatic cells (Zuluaga *et al.*, 2013). Cell wall-degrading enzymes secreted by vascular pathogens degrade the xylem walls and pit membranes of the vessels to facilitate infection and to obtain nutrients (Yadeta and Thomma, 2013). As a response, plants can build physico-chemical barriers and overaccumulate proteins and secondary metabolites in the xylem sap (Kashyap *et al.*, 2022). Proteomic-based investigations on the interaction between tomato and the vascular pathogen *Fusarium oxysporum* revealed the accumulation of various pathogenesis-related proteins (PRs) in the xylem, including β-1,3-glucanases (PR2), chitinases (PR3) and other glycosyl-hydrolases to degrade the fungal cell wall components β-1,3-glucan and chitin (Van Loon *et al.*, 2006). Other proteins secreted into the xylem included proteases, peroxidases, oxidoreductases, PR1, and PR5/5x (Rep *et al.*, 2002; Van Loon *et al.*, 2006; Houterman *et al.*, 2007; Gawehns *et al.*, 2015; De Lamo *et al.*, 2018). Interestingly, the accumulation of defence-related proteins varies depending on the level of resistance of the infected plant and the pathogen effector repertoire (Gawehns *et al.*, 2015; De Lamo *et al.*, 2018), highlighting the plasticity and controlled regulation of plant immunity.

PR1 proteins are members of the CAP protein family (cysteine-rich secretory proteins from humans, antigen 5, and pathogenesis-related 1 proteins), found in bacteria, fungi, nematodes, and plants. Most PR1 family proteins only contain the CAP domain and relatively short N- and C-terminal extensions (Gibbs *et al.*, 2008; Han *et al.*, 2023). Most PR1 proteins are thought to be secreted into the apoplastic space, via their N-terminal signal peptide, and accumulate adjacent to infected areas (Carr *et al.*, 1987; Lincoln *et al.*, 2018). PR1 proteins were first identified from leaves of *Nicotiana tabacum* infected with tobacco mosaic virus (Van Loon and Van Kammen, 1970; Durrant and Dong, 2004). Since *PR1* is strongly up-regulated as part of the salicylic acid-dependent host defence activation, it is widely used as a plant defence marker (Durrant and Dong, 2004). For instance, PR1 proteins constitute around 2% of the total leaf protein found in infected tobacco (Alexander *et al.*, 1993). Numerous PR1 family proteins were described as highly induced upon infection or treatment with plant hormones in *Solanaceae* (De Wit *et al.*, 1986; Eyal *et al.*, 1992; Van Kan *et al.*, 1992; Tornero *et al.*, 1994), but not all PR1 proteins are up-regulated during infection (Cornelissen *et al.*, 1987; Eyal *et al.*, 1992; Tornero *et al.*, 1994).

An 11-amino acid peptide derived from PR1b was reported to be highly accumulated in wounding and methyl jasmonate-treated tomato leaves (Chen *et al.*, 2014). Subsequent analysis by mass spectrometry revealed that it was originated from the C-terminus of PR1b, and it was termed CAP-derived peptide 1 (CAPE1). The CAPE peptide PxGNxxxxxPY motif is highly conserved, and the CNYx sequence upstream of the CAPE peptide was proposed as a putative cleavage motif to generate the CAPE1 peptide. Processing of PR1 in wheat is performed by an unknown serine protease (Sung *et al.*, 2021) and cleavage in Arabidopsis involves a cysteine protease with caspase-like activity (Chen *et al.*, 2023).

Impaired growth of *Phytophthora infestans* zoospores after addition of purified tomato and tobacco PR1s was reported, suggesting an antimicrobial activity (Niderman *et al.*, 1995). Secreted PR1 was shown to translocate through an unknown mechanism into *Phytophthora infestans* cells to target the AMP-activated protein kinase kinase complex, repressing growth, proliferation, and virulence (Luo *et al.*, 2023). Interestingly, the fungal pathogen *Fusarium oxysporum* f. sp. *lycopersici* (Fol) can secrete a pathogen effector, Fol-Secreted Virulence-related Protein1 (FolSvp1), which directly binds PR1, translocating from the extracellular and plasma membrane to the host nucleus (Li *et al.*, 2022). Intriguingly, several PR1s described in phytopathogenic fungi and nematodes have been shown to participate in promoting virulence and suppressing the immune response of the host (Han *et al.*, 2023).

CAP superfamily proteins have a conserved lipid binding function (Choudhary and Schneiter, 2012; Darwiche *et al.*, 2017). The sterol-binding activity of tomato PR1 proteins was found essential for their antimicrobial function *in vitro* (Gamir *et al.*, 2017), supporting the suggested role of PR1 to cause cellular leakage of fungi. However, sterols are rare in bacteria, which suggests that the antimicrobial function of PR1 against bacterial pathogens is provided by a different domain or mechanism. Finally, ectopic expression of *PR1* from crop plants in tobacco and Arabidopsis conferred increased resistance to bacterial and fungal pathogens, mediated by plant defence priming (Sarowar *et al.*, 2005; Li *et al.*, 2011; Shin *et al.*, 2014; Fang *et al.*, 2019).

The CAPE peptide of wheat was shown to suppress multiplication of the pathogenic fungus *Parastagonospora nodorum* (Sung *et al.*, 2021). Overexpression of the mulberry CAPE peptide in Arabidopsis enhanced resistance against *Botrytis cinerea* and *Pseudomonas syringae* (Fang *et al.*, 2019). Plants sprayed with CAPE peptides also showed more resistance to *Fusarium oxysporum* f. sp. *lycopersici* (Chen *et al.*, 2014; Li *et al.*, 2022). Gene expression studies indicated that multiple defence genes, including protease inhibitors, *PR7* and *PR1b*, were induced by adding exogenous CAPE1 peptide to leaves. Notably, the CAPE1 peptide did not up-regulate the expression of WRKY TRANSCRIPTION FACTOR53 (WRKY53), which is highly up-regulated by the application of the pathogen-associated molecular pattern (PAMP) peptide flg22 (Chen *et al.*, 2014). CAPE1 is a phytocytokine or a secondary damage-associated molecular pattern (DAMP), and as such, it activates different pathways from PAMPs (Koenig *et al.*, 2023). Intriguingly, CAPE peptides produced by plant pathogens in the apoplast can increase plant susceptibility by suppressing the plant’s defence responses (Lin *et al.*, 2023). Although the exact mode of action of pathogen PR1-derived CAPE peptides is not fully understood, they underscore the importance of inhibiting the signalling mechanisms activated by plant CAPEs during invasion.

We previously demonstrated that resistant tomato plants limit bacterial movement in the apoplast and the xylem (Planas-Marquès *et al.*, 2020). As a first step to dissect the underlying mechanisms, we analysed the apoplast proteome upon challenge with *R. solanacearum* in the susceptible tomato cultivar Marmande and the resistant cultivar H7996 (Planas-Marquès *et al.*, 2018). Here, we describe the xylem proteomes in these same cultivars and compare them to the apoplastic proteomes, identifying PR1s as highly induced upon infection, and characterize the role of PR1b and its derived CAPE peptide in the context of tomato infection by *R. solanacearum*.

## Materials and methods

### Plant material and growth conditions

The tomato (*Solanum lycopersicum*) lines used were the highly resistant breeding line H7996 and the highly susceptible commercial variety Marmande. For soil experiments, pots filled with Substrate 2 (Klasmann-Deilmann GmbH) mixed with perlite and vermiculite (30:1:1) were used. Plants were grown in the greenhouse at 26 °C for 4–5 weeks under long-day conditions (16 h light–8 h dark) and then transferred to a chamber set at 27 °C, 60% relative humidity and 12 h day/night photoperiod 2 d prior to inoculation with *Ralstonia solanacearum*.

For *in vitro* experiments, tomato seeds were surface sterilized in 35% bleach and 0.02% Triton X-100 for 10 min and then rinsed with sterile distilled water five times before sowing on semi-solid medium [Murashige and Skoog (MS) with agar] in square culture plates. Plates were placed standing upright in a walk-in tissue culture growth chamber set at 25 °C under long-day conditions.

*Nicotiana benthamiana* plants were grown in the greenhouse at 26 °C under long-day conditions in pots filled with sphagnum peat substrate (Gramoflor, profi-substrate, Spain). Three-week-old plants were used for infiltration with *Agrobacterium tumefaciens*.

### Bacterial material, plant inoculations, and pathogenicity assays

All assays were performed using a *R. solanacearum* GMI1000 (phylotype I, race 1, biovar 3) luminescent reporter strain carrying the *PpsbA:LuxCDABE* construct that we previously generated (Cruz *et al.*, 2014). *Ralstonia solanacearum* was routinely grown on rich B medium (10 g l^−1^ bactopeptone, 1 g l^−1^ yeast extract, and 1 g l^−1^ casaminoacids) using gentamicin (10 μg ml^−1^) for selection.

For soil-drenching infections, plants were inoculated as previously described (Planas-Marquès *et al.*, 2020). Briefly, 40 ml of a 10^8^ colony-forming units (CFU) ml^−1^ (OD_600_=0.1) bacterial suspension of *R. solanacearum* was poured on every pot after making four holes in the soil with a disposable 1 ml pipette tip. For petiole inoculation, 10 µl of a 10^6^ CFU ml^−1^ (OD_600_=0.001) suspension was pin-inoculated in the cotyledons or first internode of plants by placing a droplet of the suspension in the petiole–stem boundary and poking it through the stem with a needle (25G, BD Microlance, Becton Dickinson). Twenty-four plants were used per condition in each of three independent biological replicates. Infected plants were scored for wilting symptoms using a scale from 0 to 4, where 0=healthy plant with no wilt, 1=25%, 2=50%, 3=75%, and 4=100% of the canopy wilted. Assessment of bacterial multiplication in the roots and hypocotyls (3 cm sections) was performed by placing the respective tissues in an empty 2 ml tube and measuring the luminescence [relative light units (RLU) s^−1^] emitted by the bacteria in a luminometer (FB 12, Berthold Detection Systems). The values are given as log RLU s^−1^ g^−1^ tissue.

For bacterial multiplication in the apoplast, plants were vacuum-infiltrated with a bacterial suspension of 10^5^ CFU ml^−1^ (OD_600_=0.0001) as previously described (Planas-Marquès *et al.*, 2018). Assessment of bacterial multiplication was performed in four samples from independent plants per time point as follows: four 5 mm-diameter leaf disks were excised from each plant, the disks were ground in 200 µl sterile water-containing 1.5 ml tubes, and 10-fold dilutions were plated on rich B medium plates. CFUs were counted and bacterial growth was calculated as CFU mm^−2^ of leaf. Four replicates (*n*=12 samples) were performed but in some conditions fewer results are shown due to plate contaminations that hindered counting CFUs.

For *in vitro* infection assays, 10-day-old plantlets were pin-inoculated 1 cm below the root collar using Pasteur pipettes submerged in a 10^6^ CFU ml^–1^ (OD_600_=0.001) luminescent *R. solanacearum* suspension. Wilting symptoms were recorded and bacterial invasion visualized with a live imaging system (ImageQuant™ LAS 4000), using a 10 min exposure time with high binning setting. Three biological replicates each with 12–14 plants were performed showing similar results.

### Xylem sap collection, pooling, and concentration

Three days after inoculation, at the onset of wilt disease symptoms in susceptible Marmande, the xylem sap was collected from mock- and *R. solanacearum* petiole-inoculated Marmande and H7996 plants. For this, tomato stems were cut 5 cm above the cotyledons (inoculation point), and the first exudated droplet was carefully blotted with tissue paper. Then, plants were placed horizontally and connected to a 15 ml tube on ice, where they were allowed to bleed for 6 h. The collection system was kept in plastic boxes to maintain an elevated humidity and prevent excessive drying of the cut stems. In each experiment, the xylem sap of 6–12 plants per condition was recovered, pooled, passed through a 0.22 µm filter to get rid of any bacteria and stored at −80 °C. Once all replicates were collected, the xylem sap samples were thawed, pooled into biological replicates, and concentrated using Amicon® Ultra-15 centrifugal filter units. For each pool, 7 ml of xylem sap was concentrated to a final volume of 110–140 µl. A total of five biological replicates were obtained, each consisting on the pooling of two to three independent experiments performed on consecutive weeks. The five biological replicates were processed by in-solution trypsin digestion of proteins and analysed by liquid chromatography (LC)–tandem mass spectrometry (MS/MS).

### Proteomic analyses

#### Sample clean-up for liquid chromatography–mass spectrometry

Peptides were desalted on home-made C18 StageTips (Rappsilber *et al.*, 2007) subsequent to on-bead digestion using trypsin. The digests were passed twice over a two membrane (3M) disc StageTip. Next the immobilized peptides were washed twice with 0.5% (v/v) formic acid (FA) and eluted from the StageTips using acetonitrile [ACN; 80% (v/v)] and FA [0.5% (v/v)]. Drying was done by using a vacuum concentrator (Eppendorf, Germany) and peptide samples were resuspended in 10 µl 0.1% (v/v) FA before LC-MS.

#### Liquid chromatography–tandem mass spectrometry

Experiments were performed with an Orbitrap Elite instrument (Michalski *et al.*, 2012) (Thermo Fisher Scientific, USA) coupled to an EASY-nLC 1000 LC system (Thermo Fisher Scientific), which was operating in the one-column mode. A fused silica capillary (75 µm×35 cm) with an integrated PicoFrit emitter (New Objective, USA) packed in-house with Reprosil-Pur 120 C18-AQ 1.9 µm resin (Dr A. Maisch GmbH, Germany) was used as an analytical column. The latter was covered by a column oven (Sonation GmbH, Germany) adjusted to 45 °C during data acquisition and was attached to a nanospray flex ion source (Thermo Fisher Scientific). Peptides were directly loaded onto the analytical column with a maximum flow rate that would not exceed the pressure limit of 980 bar (usually around 0.6–0.8 µl min^−1^). Peptides were subsequently separated on the analytical column by running a 140 min gradient of solvent A (0.1% FA in water) and solvent B (0.1% FA in ACN), both of UPLC grade (Sigma, Germany), at a flow rate of 300 nl min^−1^ (gradient: start with 7% B; gradient 7–35% B for 120 min; gradient 35–100% B for 10 min, and 100% B for 10 min). The software Xcalibur (Thermo Fischer Scientific, UK; version 2.2 SP1.48) was used to operate the mass spectrometer in the positive ion mode. Precursor ion scanning was performed in the Orbitrap analyser (Fourier transform mass spectrometry) in the scan range of *m*/*z* 300–1,800 at a resolution of 60 000 with the internal lock mass option (lock mass was 445.120025 *m*/*z*, polysiloxane) (Olsen *et al.*, 2005) turned on. Within the ion trap (ion trap mobility spectrometry), ion spectra were recorded in a data-dependent manner at a rapid scan rate using a variable scan range. We set the spray voltage (ionization potential) to 1.8 kV. Analysis of peptides was performed by using a repeating cycle consisting of a full precursor ion scan (3.0×10^6^ ions or 50 ms) followed by 15 product ion scans (1.0×10^4^ ions or 50 ms). Peptides were isolated based on their intensities in the full survey scan (threshold of 500 counts) for MS/MS generation allowing peptide sequencing and identification. During the generation of MS/MS spectra the collision-induced dissociation energy was set to 35%. For acquisition of MS/MS data, a dynamic ion exclusion of 120 s with a maximum list of excluded ions consisting of 500 members and a repeat count of 1 was used. Furthermore, the preview mode for the orbitrap, ion injection time prediction, monoisotopic precursor selection, and charge state screening were enabled. Charge states ≤1 were not considered for fragmentation.

#### Peptide and protein identification

The software MaxQuant (version 1.5.3.30) was used and raw MS/MS spectra were submitted to an Andromeda (Cox *et al.*, 2011) search at default settings (Cox and Mann, 2008) and activated label-free quantification (Cox *et al.*, 2014). Andromeda searches allowed us to include oxidation of methionine residues (16 Da) and acetylation of the protein N-terminus (42 Da) as dynamic modifications and static modification of cysteine (57 Da, alkylation with iodoacetamide). Spectra data were searched against the Uniprot *Ralstonia solanacearum* (strain GMI1000) reference proteome database (UP000001436_267608.fasta; 5002 entries; downloaded 24 May 2018) and the Uniprot reference database for *Solanum lycopersicum* (UP000004994_4081; 33952 entries, downloaded 24.05.2018). A database (containing known MS contaminants as implemented in MaxQuant, 245 sequences) was included to estimate the level of contamination. The instrument type in Andromeda searches was set to Orbitrap and the precursor mass tolerance to ±20 ppm (first search) and ±4.5 ppm (main search). A value of ±0.5 Da was used for the MS/MS match tolerance, while the enzyme specificity was set to ‘Trypsin/P’. Both the peptide spectrum match false discovery rate (FDR) and the protein FDR were set to 0.01 (based on the target–decoy approach). For protein quantification unique and razor peptides at a minimum peptide length of seven amino acids were allowed. Modified peptides with a minimum score of 40 and with dynamic modifications were allowed for quantification. Filtering of the MaxQuant output and further data analysis were performed using the software Perseus v1.6.1.3 (Tyanova *et al.*, 2016). MS/MS counts were loaded into the matrix from the proteinGroups.txt file. Generally, all potential contaminants and reverse hits were removed. The same was done with hits only identified by site. For statistical calculations technical replicates were integrated in categorial groups and filtered. Only protein groups containing three valid values in a minimum of one categorical group were further processed. Student’s *t*-test depicted in volcano plots was performed (number of randomizations=250; initial FDR=0.05/0.01 and S0=0.1).

### Sequence comparison and phylogenetic analyses of PR1 proteins

BLASTn/p searches to identify new tomato *PR1* genes and putative orthologues were performed on the Sol Genomics (SGN) database (www.solgenomics.net), the NCBI databases (https://www.ncbi.nlm.nih.gov/), and UniProt (https://www.uniprot.org/) when indicated. To identify the tomato PR1s, the NCBI searches were performed using the ‘Nucleotide collection (nr/nt)’ database, specifying ‘*S. lycopersicum* (taxid: 4081)’ as the organism. The species’ specific databases used to identify putative PR1 orthologues are indicated in Supplementary Table S1, and the equivalence between gene and protein identifiers of tomato PR1s in the SGN, NCBI, and UniprotKB databases is indicated in Supplementary Table S2.

Neighbour-joining trees and amino acid sequence identity matrices were constructed from the respective ClustalO sequence alignments (https://www.ebi.ac.uk/Tools/msa/clustalo/). Trees were visualized by the Interactive Tree of Life (iTOL) webtool (https://itol.embl.de). Alignment analysis was performed by MEGA-X with the ClustalO method and visualized by Java Tree.

### Cloning of the PR1 proteins

All primers used for cloning are detailed in Supplementary Table S3. For transient expression in *N. benthamiana*, the open reading frame of PR1b was amplified from the tomato H7996 line by PCR. Six histidine codons were included in the reverse PCR primers to facilitate purification of the resulting recombinant proteins. PCR products were cloned into pJET1.2/blunt (Thermo Fisher Scientific) and amplified in *Escherichia coli* TOP10. The insert was cloned in between the CaMV 35S promoter and terminator of pART7 (Gleave, 1992) using the restriction enzymes *Sma*I and *Bam*HI. The expression cassettes were then transferred to the binary vector pART 27 (Gleave, 1992) using *Not*I. The vectors were ultimately introduced into *Agrobacterium tumefaciens* strain C58C1 for transient expression in *N. benthamiana*.

For generation of overexpression *PR1b* lines, the open reading frames (ORFs) of *PR1b* and PR1b^ΔCAPE^ driven by the 35S promoter were amplified from the pART7-PR1b by PCR. An haemagglutinin (HA)-tag was inserted between N-terminal signal peptides as determined by SignalP (Almagro Armenteros *et al.*, 2019) and the CAP domain by overlapping PCR. The final fragments were cloned into pART27 vectors with *Not*I restriction enzyme.

For purification of PR1b proteins, ORFs of PR1b^fl^ and PR1b^ΔCAPE^, lacking their N-terminal signal peptides, were amplified from the tomato H7996 line by PCR. PCR products were cloned into pJET1.2/blunt and amplified in *E. coli* TOP10. For generation of PR1b^KK^, based on pJET1.2- PR1b^fl^ site, the QuikChange II Site-Directed Mutagenesis Kit (Agilent) was used. The 148th tyrosine and 149th aspartic acid residues of PR1b were replaced with lysine. Inserts were then cloned in the multiple cloning site of pCold using the restriction enzymes *Nde*I and *Hin*dIII.

### Transient expression PR1 proteins in *Nicotiana benthamiana*

Transient expression in *N. benthamiana* was performed as previously described (Reichardt *et al.*, 2018). Briefly, *A. tumefaciens* ASE containing pSoup and the PR1s and PR1s-PR1b^ΔCAPE^ expression construct in pART27 or the p19 suppressor of silencing was grown on LB plates with appropriate antibiotics. Bacteria were collected from the plate with a 1 ml disposable pipette tip and resuspended in 10 ml infiltration buffer (10 mM MES pH 5.6, 10 mM MgCl_2_). Constructs were mixed to a final OD_600_ of 0.3 for C58C1 carrying pART27-PR1s and 0.3 for C58C1 carrying p19 and infiltrated into fully expanded leaves of a 3- to 4-week-old *N. benthamiana* using a blunt syringe.

### Generation of stable *PR1b* overexpression tomato plants

pART27 containing 35S::PR1b-HA or 35S:: PR1b^ΔCAPE^-HA was transformed into Marmande tomato plants. For this, the constructs were transformed into *A. tumefaciens* strain C58C1. Cotyledon explant preparation, selection, and regeneration followed the methods described by Mazier *et al.* (2011). Transformants were selected on kanamycin-containing medium. Accumulation of PR1b and PR1b^ΔCAPE^ protein was assayed by immunoblot with a monoclonal HA antibody (GenScript, Piscataway, NJ, USA).

### Purification of PR1b proteins and derivatives

The PR1 proteins cloned in the P-COLD vector were expressed in *E. coli* Rosetta-competent cells (Lobstein *et al.*, 2012). Cells containing the target construct were grown in 50 ml LB medium with ampicillin at 37 °C on a shaker overnight, transferred to 450 ml fresh LB medium with ampicillin for another 3 h, then 200 mM isopropyl β-d-1-thiogalactopyranoside was added before transferring the flask into a 16 °C shaker overnight. The 500 ml cell culture was resuspended in 50 ml lysis buffer consisting of 50 mM HEPES pH 8.0 and 500 mM NaCl, and lysed by two sequential passes through a French press cell at around 15 000–20 000 psi. The lysate was then clarified by centrifugation at 12 000 *g* at 4 °C for 30 min. After passing the lysate through a 0.45 µm filter, the proteins of interest were separated by using immobilized metal affinity chromatography (IMAC) (His-trap 5 ml), facilitated by the presence of an N-terminal cleavable six-histidine tag. The proteins were eluted from the IMAC column with the elution buffer (50 mM Hepes pH 8.0, 500 mM NaCl, and 300 mM imidazole). After elution, excess imidazole was removed by a 10 kDa molecular weight cutoff (MWCO) ultrafiltration device (Vivaspin 500).

To remove the ProS2 domain and the histidine tag, PR1 fractions were treated with the P69 protease (Zhang *et al.*, 2024) for 3 h at room temperature, leaving a 22-residue N-terminal overhang (Ser–Asp–Ala). The cleaved solution was reapplied to the Ni-nitrilotriacetic acid (NTA) beads to remove His-ProS2, His-P69, and uncleaved target proteins. Fractions corresponding to the PR1b proteins were collected and visualized by Coomassie-stained SDS-PAGE, before being concentrated using the 10 kDa MWCO ultrafiltration device (Vivaspin 500) for further analysis.

### Generation of CRISPR/Cas9 *pr1b* mutant tomato plants

The CRISPR/Cas9 vectors and the cloning methodology used in this study was described by Danilo *et al.* (2018). Briefly, small guide (sg)RNA sequences targeting the *PR1b* gene were designed based on high specificity score and the lowest number of off-target genes on the CRISPOR web tool (Haeussler *et al.*, 2016; http://crispor.tefor.net/; Supplementary Table S4). sgRNA sequences under the control of the U3 or U6 sgRNA promoter were synthesized flanked by gateway recombination sites (Danilo *et al.*, 2018). sgRNA cassettes were introduced into pDONR207 by BP Gateway recombination. To generate deletions in each particular gene (Supplementary Table S4), U3- and U6-sgRNA cassettes were combined into single vectors. For this, the U6-sgRNA cassette targeting one specific gene was excised from pDONR207 with *Xho*I and *Pst*I and mobilized into the U3-sgRNA cassette-containing pDONR207 plasmid previously digested with *Sal*I and *Pst*I. The resulting vectors (pDONR207-U3-sgRNA-U6-sgRNA) were then introduced by LR Gateway recombination into the binary vectors pDe-Cas9-*NptII* or pDe-Cas9-*Hpt* described in Danilo *et al.* (2018) for later selection with kanamycin.

For tomato H7996 genotype and Marmande genotype transformation, the constructs were transformed into *A. tumefaciens* strain C58C1. Cotyledon explant preparation, selection, and regeneration followed the methods described by Mazier *et al.* (2011). Transformants were selected on kanamycin-containing medium. Successfully regenerated CRISPR/Cas9 plantlets were screened by PCR. For this, leaf samples were collected and genomic DNA was extracted by cetyltrimethylammonium bromide (CTAB) buffer. Deletions were screened using the genotyping primers listed in Supplementary Table S3 and confirmed by Sanger DNA sequencing.

### Extraction of apoplast and total extract from tomato leaves and *N. benthamiana* leaves

For extraction of apoplastic fluid of tomato leaves, full-size true leaves were excised, immersed in 50 ml of cold distilled water and infiltrated by vacuum for 1 min. After vacuum, leaves were surface-dried with paper, wrapped in 5 ml cut-tip pipette tips, and placed side up in a 50 ml centrifuge tube. After centrifugation (3000 *g*, 10 min, 4 °C), around 300 μl of apoplastic fluid was harvested. The apoplastic fluid was finally passed through a 0.22 µm filter to get rid of any bacteria, diluted to 1 mg ml^−1^, protein concentration was measured by Protein Assay Dye Reagent (Bio-Rad), and the fluid was stored at −80 °C.

For total extract isolation, the main vein was cut from whole leaves, and the rest of the leaf was quickly frozen in liquid nitrogen. After grinding, leaf material was mixed with 5 ml of G-TEN buffer [10% glycerol, 100 mM Tris–HCl pH 7.5, 1 mM EDTA, 150 mM NaC and 1× protease inhibitor cocktail (Sigma, P599)] and centrifuged for 20 min at 10 000 *g* at 4 °C. After centrifugation, the supernatant was passed through Miracloth (EMD Millipore Corp.) to filter debris. Laemmli sample buffer (5×) was added to 100 µl of filtrate and boiled for 5 min. Equal amounts of supernatant were loaded on 12% SDS-PAGE gels.

### Confocal microscopy

For live-cell imaging, infiltrated parts of *N. benthamiana* leaves were used for observation under the Olympus FV1000 confocal microscope using a ×60 water immersion objective. Excitation wavelengths used were 488 nm for green fluorescent protein (GFP) and 561 nm for red fluorescent protein (RFP). The images were analysed using ImageJ.

### Treatment plants with CAPE1 peptide

For checking the antimicrobial activity of CAPE1 peptide in plants and its effect on expression of hormone-related genes involved in plant defence, the aerial part of 4-week-old tomato plants was sprayed with 500 nM CAPE1 peptide for 3 h in a plastic box to maintain high humidity. Then plants were vacuum-infiltrated with a bacterial suspension, and RNA was extracted for subsequent quantitative reverse transcription–PCR (qRT-PCR) at 3 h or 6 h post-inoculation. Three technical PCR replicates were performed from three samples obtained in independent biological replicas.

### Antimicrobial assay *in vitro*

Testing for antimicrobial activity of recombinant PR1 proteins was carried out as described by Gamir *et al.* (2017). Briefly, *R. solanacearum* at 10^8^ CFU ml^−1^ (OD_600_=0.1) in a total volume of 200 μl rich B medium with different concentrations of CAPE1 peptide and recombinant PR1b and derivatives was incubated in a 96-well microtitre plate (Greiner). Bacterial growth was recorded over time in >10 independent cultures using a VICTOR Nivo device (PerkinElmer).

### RNA extraction and quantitative reverse transcription–PCR analysis

The Maxwell RSC Plant RNA kit (Promega) was used to isolate RNA from leaves from three different plants. RNA (2 µg) was reverse transcribed into cDNA with the Applied Biosystems High-Capacity cDNA Reverse Transcription Kit with RNase inhibitor (Thermo Fisher Scientific). RT-qPCRs were performed with LightCycler SYBRgreen I master (Roche) in a LightCycler 480 System (Roche). All gene expression was normalized to expression of *SlACT2* (Solyc11g005330.1) and data were analysed using the ΔΔ*C*_T_ method. Invariant expression of the reference *SlACT2* gene under all conditions studied was verified (Supplementary Fig. S1) using both ANOVA and paired Student’s *t*-test analyses of the *C*_t_ values in each condition. Primers for RT-qPCR used in this study were previously described and are listed in Supplementary Table S3.

### Statistical analyses

Statistical analyses were performed using the corresponding R package. All statistical tests are indicated in the respective methods or figure legends.

## Results

### Xylem sap and apoplast proteomes reveal high accumulation of PR1 proteins upon *R. solanacearum* infection

We previously showed that the active protease landscape of tomato undergoes significant changes after being challenged with *R. solanacearum* (Planas-Marquès *et al.*, 2018). This response might be at play during the initial stages of the infection, when *R. solanacearum* traverses the root epidermis and cortex before reaching the xylem vessels. However, additional defence mechanisms have been reported in the xylem of resistant tomato plants (Grimault *et al.*, 1994*b*; McGarvey *et al.*, 1999; Nakaho *et al.*, 2000, 2004; Planas-Marquès *et al.*, 2020; Kashyap *et al.*, 2022), which explain the plants’ ability to hamper bacterial colonization within this tissue (Planas-Marquès *et al.*, 2020). To further characterize these defence mechanisms, we decided to investigate the proteomic changes that occurred in the xylem sap of susceptible and resistant tomato plants upon *R. solanacearum* infection.

**Fig. 1.**
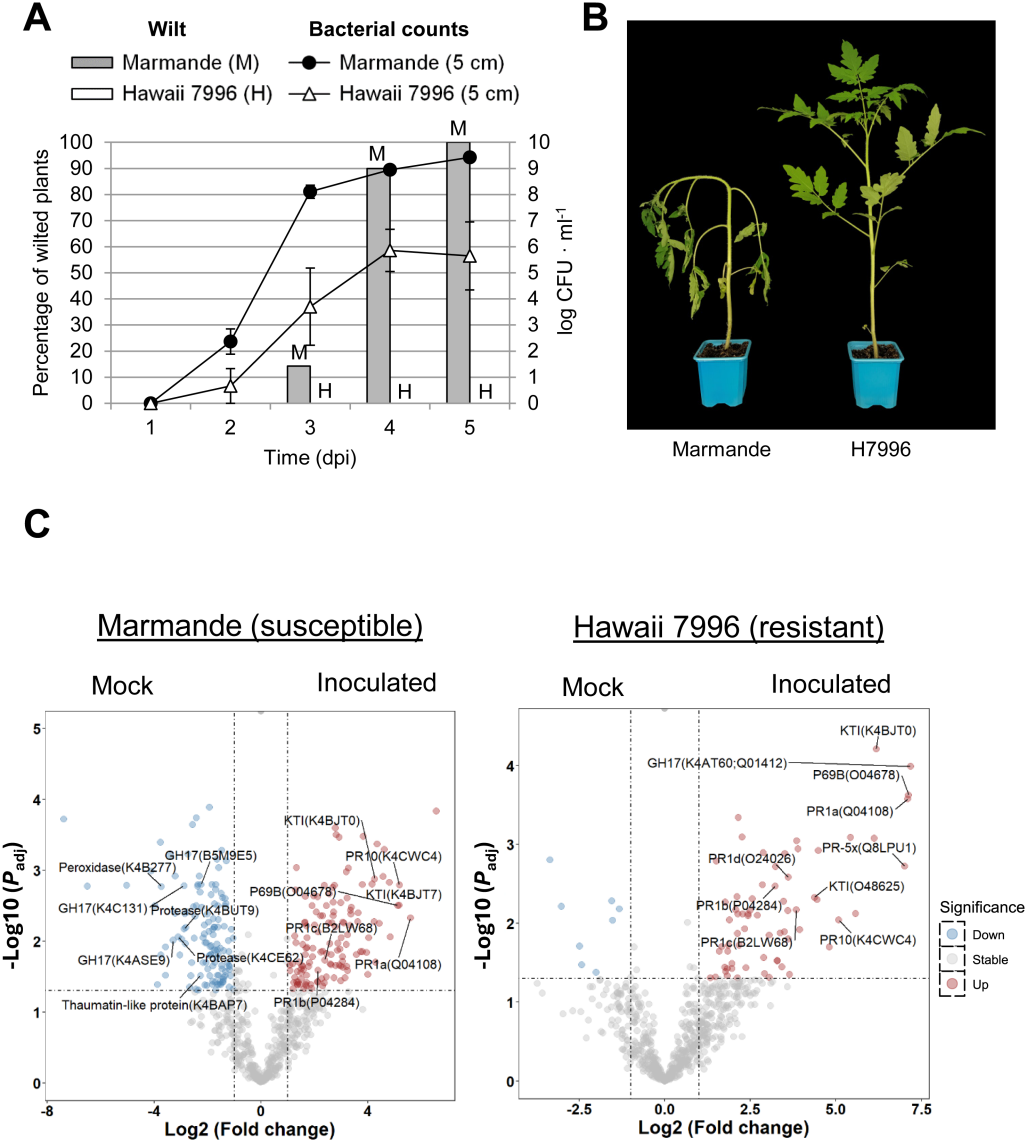
*Ralstonia solanacearum* infection causes differential proteomic changes in susceptible (Marmande) and resistant (Hawaii 7996, H7996) tomato varieties. Four- to five-week-old Marmande and H7996 tomato plants were petiole-inoculated with a luminescent *R. solanacearum* reporter strain. (A) Wild disease symptoms and bacterial loads were monitored over time. (B) Representative picture of Marmande and H7996 plants 5 d post-inoculation. (C) Volcano plots summarizing the proteomics analysis. Logarithm of the adjusted *P*-values plotted against the logarithm of fold change (FC) of the conditions indicated in each graph. Left and right panel indicate the comparison of mock and *R. solanacearum* inoculation in susceptible Marmande or resistant H7996. Dashed lines indicate adjusted *P*-value=0.05, and FC=2 or 0.5. PR1, pathogenesis-related 1; GH17, glycoside hydrolase 17; KTI, Kunitz trypsin inhibitor.

For this, susceptible (Marmande) or resistant (H7996) tomato plants were petiole-inoculated with *R. solanacearum* GMI1000 or mock and 3 days post-inoculation (dpi) xylem sap was collected. Petiole-inoculation was chosen as the inoculation method instead of a natural soil-drench assay to avoid sample variability derived from the intrinsic stochasticity of root colonization and to ensure higher/detectable amounts of bacteria in the stems. Samples were taken at 3 dpi given that at this time point bacteria are readily detectable and susceptible plants are just starting to show disease symptoms (Fig. 1A, B). Xylem sap proteins from five biological replicates per condition were then analysed by mass spectrometry, yielding a total of 695 tomato protein groups after filtering out non-robust detections (Supplementary Dataset S1). A principal component analysis (PCA) plot of the filtered and normalized data showed sample clustering by condition (Supplementary Fig. S2). In terms of protein identity, the two proteomes were very similar (80%) in mock conditions but differed 8% upon infection (Supplementary Fig. S3B). Interestingly, a slight increase in change (11%) was detected in susceptible plants upon infection compared with the resistant H7996 cultivar (Supplementary Fig. S3B), which correlated with the number of differentially accumulated proteins (DAPs) that resulted from the statistical analysis (Supplementary Fig. S3C). Hence, the Marmande tomato variety experienced more pronounced proteomic changes than H7996. This behaviour was particularly noticeable in the number of reduced proteins upon *R. solanacearum* infection. On the other hand, a significant higher number of increased proteins were detected in H7996 when comparing the two infected xylem sap proteomes.

We then examined the identity of the DAPs between the two tomato varieties in mock conditions and upon infection (Fig. 1C). The PR protein PR5x was one of the most increased proteins upon infection (235-fold higher in H7996 compared with Marmande). Some proteins were similarly accumulated in the xylem sap of Marmande and H7996, including various PR proteins, as well as the subtilisin-like protease P69B and several Kunitz-type trypsin inhibitors (KTI). However, a major decrease in protein accumulation upon infection was only observed in the susceptible variety and included PR proteins from the thaumatin-like superfamily, peroxidases, glycoside hydrolases (GHs) and various proteases and peptidases (Fig. 1C). Interestingly, among the GHs, three GH17s were identified as less accumulated in Marmande (B5M9E5, K4C131, and K4ASE9) (Fig. 1C).

**Fig. 2.**
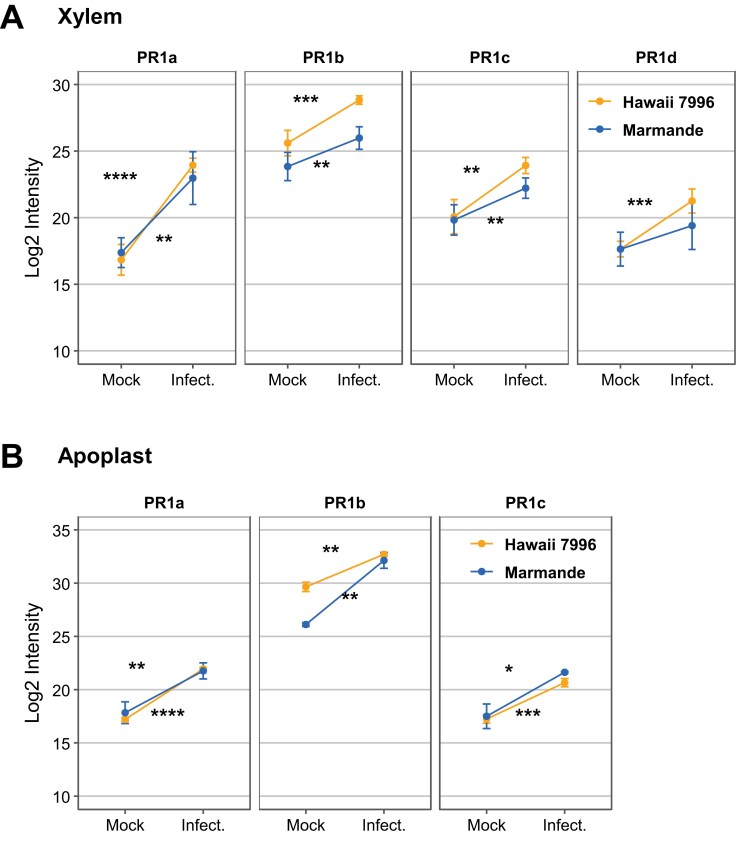
PR1 protein levels change in response to infection with *R. solanacearum*. Log2 abundance intensity of PR1s of Marmande and Hawaii 7996 upon mock treatment and *R. solanacearum* infection in the xylem (A) and apoplast (Planas-Marquès *et al.*, 2018) (B). Error bars indicate standard error of three biological replicates. Asterisks indicate activity or abundance significant differences on infection by Student’s *t*-test (*P<0.05, **P<0.01, ***P<0.001, ****P<0.0001).

In order to identify common responses that might act as immunity hubs against *R. solanacearum*, we compared the xylem sap and the apoplastic fluid proteomes (Fig. 1; Planas-Marquès *et al.*, 2018). Interestingly, we found that four PR1s were increased in response to *R. solanacearum* infection in the xylem of resistant H7996 and susceptible Marmande plants (Fig. 2A), while three PR1s highly accumulated in the apoplast of both resistant and susceptible plants (Fig. 2B). Notably, in the xylem, PR1s are more abundant in H7996 than in Marmande (Fig. 1). Comparison of both proteomes upon infection also revealed KTIs as the most responsive proteins (Supplementary Fig. S3).

### Characterization of the tomato PR1 family and its conservation

Four *PR1* genes had been identified in tomato before the tomato genome was publicly available and they were named following different nomenclature systems in the literature (Eyal *et al.*, 1992; Tornero *et al.*, 1994; Niderman *et al.*, 1995). To identify all tomato *PR1* genes and name them consistently, the coding sequence of *PR1b* (Solyc00g174340) was used as bait in a BLASTn on the Sol Genomics Network and the NCBI databases. Ten highly similar genes (>82% sequence identity) were retrieved, four of which corresponded to the previously reported PR1 proteins. A neighbour-joining tree of the encoded amino acid sequences, including the closest non-PR1 in tomato (Solyc02g065470.1.1) as an outgroup, revealed that PR1a, b, and d are the most closely related (Supplementary Fig. S4).

**Fig. 3.**
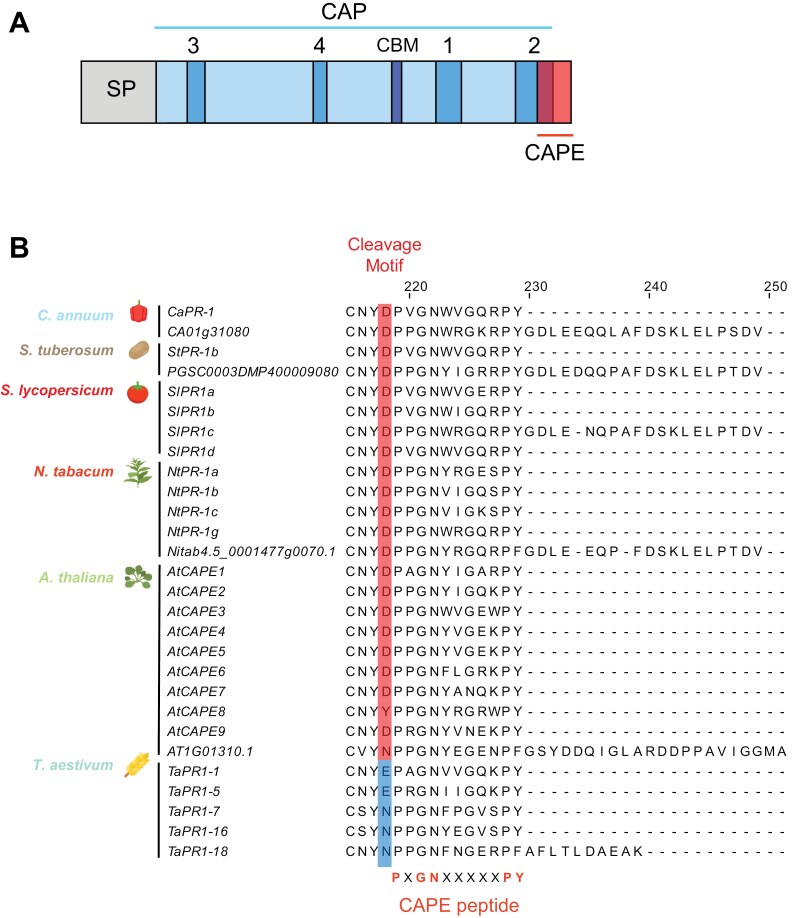
PR1 protein features and alignment. (A) Representation of the tomato PR1 protein domains and conserved features. SP, signal peptide; CAP, cysteine-rich secretory proteins, antigen 5, and pathogenesis-related 1 protein domain, highlighting CAP3, CAP4, CAP1, CAP2, and CBM motifs; CAPE, CAP-derived peptide (Han *et al.*, 2023). (B) Alignment of the representative PR1 proteins across different plant species. Amino acid alignment generated from ClustalO alignment of the representative PR1 proteins from tomato (*Solanum lycopersicum*), potato (*Solanum tuberosum*), pepper (*Capsicum anuum*), tobacco (*Nicotiana tabacum*), Arabidopsis (*Arabidopsis thaliana*), and wheat (*Triticum aestivum*). Red and blue highlighted regions show the last amino acid before the putative CAPE peptide cleavage. The conserved CAPE peptide sequence is shown in red.

Protein alignment revealed that most tomato PR1s featured the predicted N-terminal signal peptide absent in the mature protein, a CAP domain, and the CAPE peptide (Fig. 3; Supplementary Fig. S4A). The putative catalytic cysteine and the predicted cleavage site before the CAPE peptide were conserved in all PR1s. CAPE peptide sequences were not identical and PR1c showed a short amino acid extension after the CAPE peptide (Fig. 3B; Supplementary Fig. S4A). Alignment of representative PR1s from different species showed that the cleavage site (CNYD) is highly conserved in *Solanaceae* and Arabidopsis, indicating a common CAPE cleavage mechanism, while the cleavage motif of wheat diverges (Fig. 3B).

To uncover the subcellular localization of tomato PR1s, we cloned the coding sequence of *PR1b* and its uncleavable version *PR1b*^*CNAD*^ fused to *GFP*. After transient expression in *N. benthamiana* and fluorescence microscopy analysis, we observed that PR1b–GFP showed a pattern compatible with its localization at the multivesicular body (MVB), while the PR1b^CNAD^–GFP localized to the endoplasmic reticulum (Supplementary Fig. S5). These data confirmed previous studies performed in Arabidopsis with AtPR1 (Pečenková *et al.*, 2017), indicating that PR1s may be secreted to the apoplast through the endoplasmic reticulum/Golgi/MVB secretory pathway, and its cleavage site plays a key role in translocation of PR1s from endoplasmic reticulum to MVB.

### PR1b involvement in tomato defence

Since PR1b was the most abundant PR1 protein in both the apoplast and the xylem (Chen *et al.*, 2014; Li *et al.*, 2022), we concentrated on this family member to study the role of PR1s in the interaction between tomato and *R. solanacearum*. We generated PR1b-defective tomato cv Marmande by CRISPR/Cas9 using two sgRNAs simultaneously (Supplementary Fig. S6A). Deletions in *PR1b* were detected only in 2 out of 63 transgenic plants obtained. After selfing, we confirmed that both lines carried the same 112 bp deletion in homozygosity without Cas9 and named them ΔPR1b-1 and ΔPR1b-2. The frameshift deletion generated an early stop codon and a truncated protein lacking the CAPE1 peptide (Supplementary Fig. S6B, C). Tomato cv Marmande plants overexpressing the full length PR1b (PR1b OE) or PR1b devoid of the CAPE1 peptide (PR1b^ΔCAPE^ OE), both including an HA-tag after the N-terminal signal peptide, were also obtained (Supplementary Fig. S7). Two independent lines overexpressing PR1b^ΔCAPE^ were used for further characterization but only one for the full length, as this was the only one found to consistently overexpress *PR1b* over generations.

**Fig. 4.**
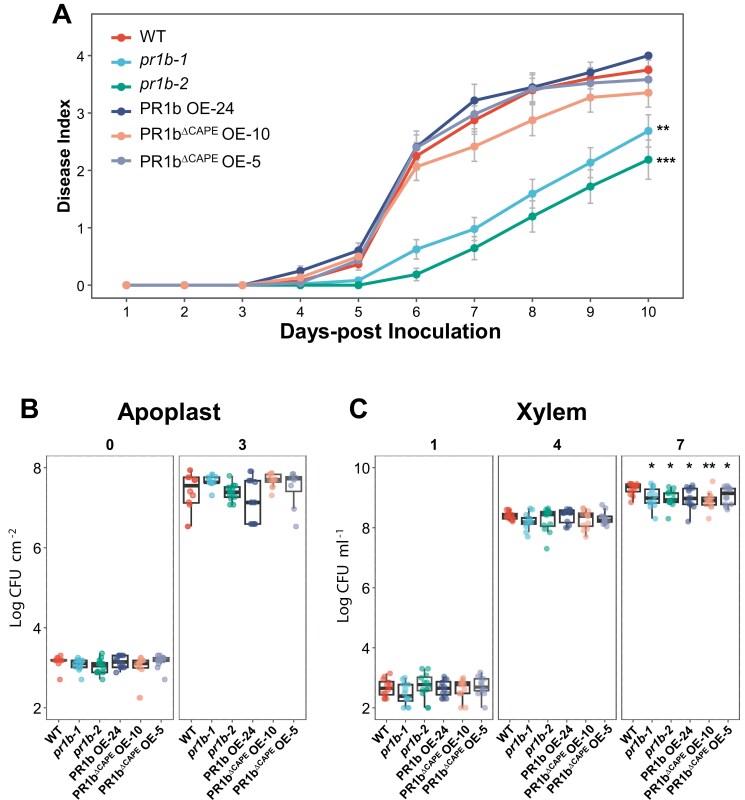
Lack of PR1b results in enhanced resistance to *R. solanacearum*. (A) Pathogenicity assay comparing PR1b mutant and overexpressing lines. Four- to five-week-old Marmande and H7996 tomato plants were soil-drenched with a 10^7^ colony-forming units (CFU) ml^−1^  *R. solanacearum* suspension. Wilting symptoms were scored over time for 2 weeks. At least 20 plants of each variety and line were inoculated. The experiment was repeated at least three times, and a representative experiment is shown. Asterisks indicate statistically significant differences between wild type and each of the *pr1b* mutant lines analysed using a paired Student’s *t*-test (**P<0.01, ***P<0.001). (B, C) Bacterial multiplication in the leaf apoplast (B) and the xylem (C). The aerial part of 4-week-old tomato plants was vacuum-infiltrated with a 10^5^ CFU ml^−1^ suspension of *R. solanacearum*, and bacterial loads were evaluated over time. Six biological replicates were assayed per day and plant genotype. Each dot corresponds to a biological replica from an independent leaf. Significant differences to wild type are shown (*P<0.05, **P <0.01, Student’s *t*-test).

Soil drench inoculation with *R. solanacearum* showed that PR1b-deficient plants were slightly more resistant than the wild type Marmande, while overexpressors displayed no differences from Marmande (Fig. 4A). To investigate if PR1b influenced bacterial multiplication in the apoplast or the xylem, the bacterium was vacuum-infiltrated in leaves or pin-inoculated in the stem, respectively, and bacterial loads were recorded over time. Earlier time points were used for growth in the apoplast, since apparent wilting symptoms appear in the leaves 4 d after infiltration. No significant differences on *R. solanacearum* multiplication in the apoplast were observed between wild type and the *PR1b* mutant or overexpressing lines (Fig. 4B). Interestingly, both the overexpression of the different *PR1b* variants and mutation of the gene caused a reduction in bacterial growth in the xylem (Fig. 4C).

To test if PR1b or its CAP domain had antimicrobial capacities, *R. solanacearum* was grown in rich medium supplemented with recombinant PR1b. Purified PR1b, PR1b^ΔCAPE^, and PR1b^KK^, the latter bearing a mutated CAPE cleavage site (CNYD to CNKK; Supplementary Fig. S8), were used. None of these PR1b variants affected *R. solanacearum* growth (Supplementary Fig. S8), ruling out their potential antimicrobial activity against *R. solanacearum*.

### Role of the CAPE1 peptide in response to *R. solanacearum*

**Fig. 5.**
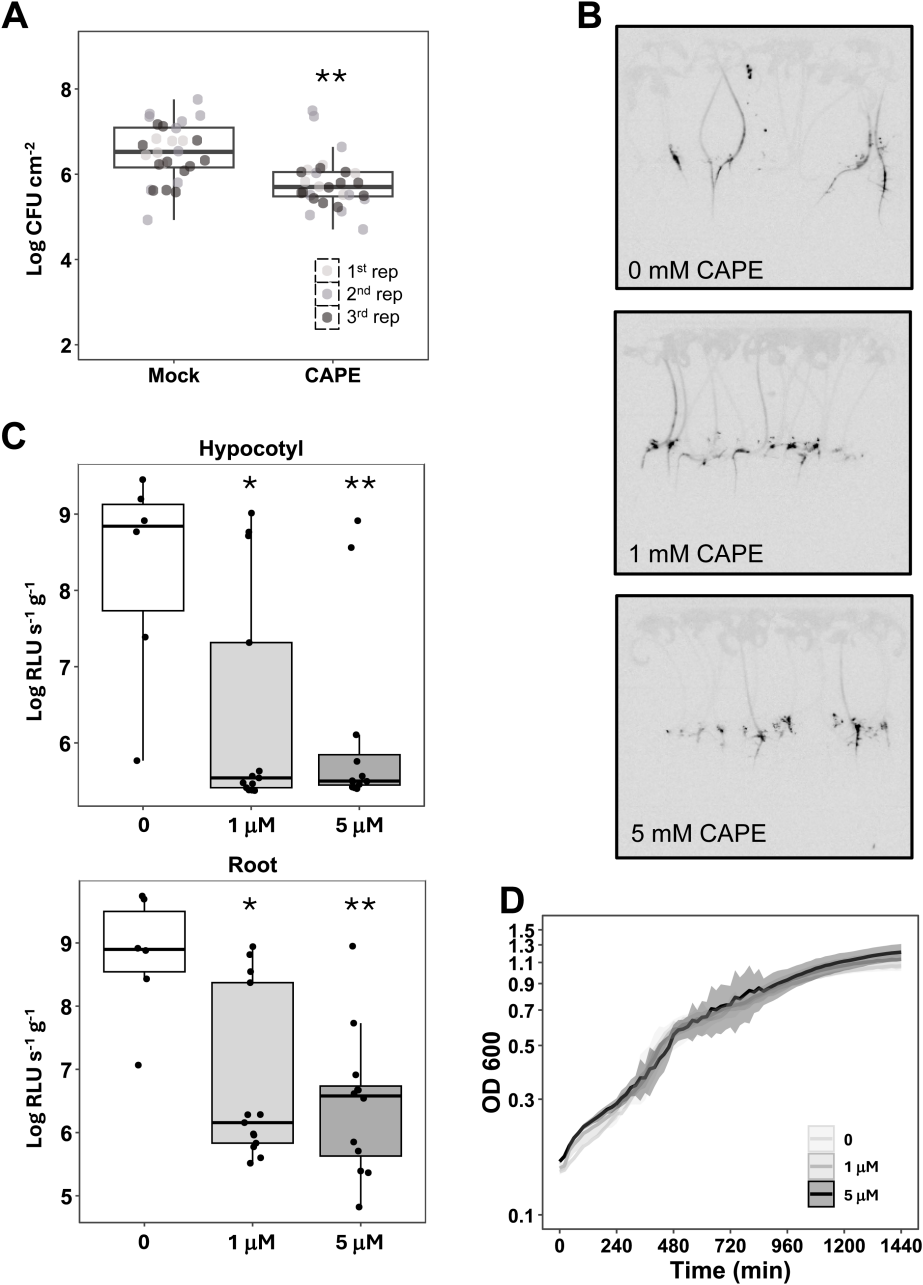
The CAPE1 peptide has an antimicrobial function against *R. solanacearum* in tomato plants. (A) The aerial part of 4-week-old Marmande tomato plants was sprayed with 500 nM CAPE peptide and 3 h later vacuum-infiltrated with a 10^5^ CFU ml^−1^ suspension of *R. solanacearum*; bacterial loads were evaluated after 3 d of infection. Three biological replicates were performed; asterisks indicate significant differences with and without CAPE peptide treatment using a *t*-test (**P<0.01). (B) Marmande seedlings were grown on MS plates containing different concentrations of CAPE1 peptide, and 10 d later they were pin-inoculated in the taproot with a luminescent *R. solanacearum* strain. A representative photograph is shown for each treatment at 7 d post-inoculation. (C) Bacterial loads in the hypocotyl and root of the plants in (B) were calculated based on the luminescence signal and are expressed as log CFU g^−1^ tissue. Three biological replicates were performed and at least six plants were measured by luminescence. Asterisks indicate significant differences with and without the CAPE1 peptide treatment using a *t*-test (*P<0.05, **P<0.01). (D) Growth of *R. solanacearum* in rich medium adding the indicated concentrations of the CAPE1 peptide. The highlighted area corresponds to the standard deviation. Three biological replicates were performed.

To understand the function of the PR1b-derived CAPE1 peptide, we chemically synthesized it and analysed its antimicrobial activity *in planta.* The peptide (500 nM) or water as a control was sprayed on the leaf surface of susceptible tomato plants (Marmande), *R. solanacearum* was vacuum-infiltrated into the leaves 3 h later, and bacterial counts measured at 3 dpi. As shown in Fig. 5A, CAPE1 treatment significantly reduced *R. solanacearum* multiplication. To address whether this effect on pathogen growth could protect plants from *R. solanacearum*, we drop-inoculated a luminescent strain of the pathogen in the taproot of plants axenically grown on plates supplemented or not with the CAPE1 peptide. At 7 dpi, *R. solanacearum* had spread both gravitropically (root) and antigravitropically (hypocotyl) from the infection site (Fig. 5B). In comparison, *R. solanacearum* spread less on both hypocotyls and roots of plants grown on medium supplemented with the CAPE1 peptide in a dose-dependent manner (Fig. 5B). In plants grown on medium supplemented with 5 µM CAPE1 peptide, the bacterium remained confined at infection sites, and it was unable to spread in the plant tissues (Fig. 5B). This observation was underscored by the reduction of bacterial loads in the hypocotyl and root of infected plants pretreated with CAPE1 (Fig. 5C). These data demonstrate that the CAPE1 peptide effectively reduces proliferation of *R. solanacearum* in root and hypocotyl of tomato plants. To determine whether CAPE1 directly affected growth of *R. solanacearum*, we added increasing concentrations of the peptide in rich medium and measured bacterial growth over time. As shown in Fig. 5D, CAPE1 peptide did not affect *R. solanacearum* growth. Together, these results show that the CAPE1 peptide can only affect multiplication of *R. solanacearum in planta*, but not when the bacterium is grown in medium, suggesting an antimicrobial function through triggering plant immunity.

**Fig. 6.**
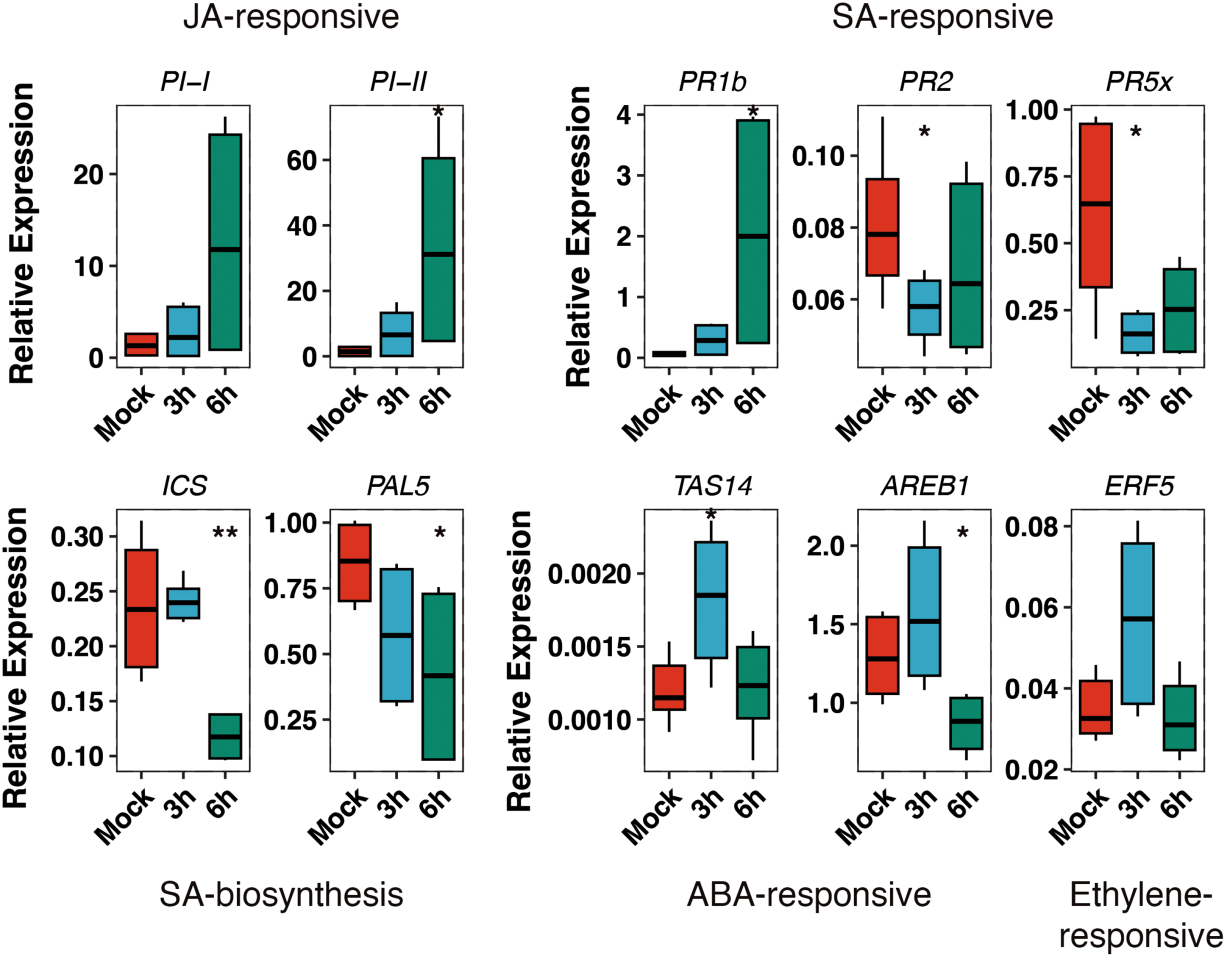
CAPE1 peptide treatment results in defence gene reprogramming in tomato. The aerial part of 4-week-old Marmande tomato plants was sprayed with water or 500 nM CAPE1 peptide for 3 h and RNA was extracted to check expression of hormone-related genes involved in plant defence was analysed by. The Actin gene (*SlAct2*) was used as endogenous reference. One disk of a CAPE1- or water-sprayed leaf was collected after 3 and 6 h for RNA extraction and cDNA synthesis. ABA, abscisic acid; JA, jasmonic acid; SA, salicylic acid.

The CAPE1 peptide derived from PR1b has been shown to prime plant defence in tomato (Chen *et al.*, 2014). Thus, we analysed expression of defence priming marker genes in tomato leaves after CAPE1 peptide exposure over time (Chen *et al.*, 2014). We measured the expression of jasmonic acid (JA)-responsive genes (*PI-I* and *PI-II*), salicylic acid (SA)-responsive genes (*PR1b*, *PR2*, and *PR5x*), abscisic acid (ABA)-responsive genes (*TAS14* and *AREB1*), and an ethylene-responsive gene (*ERF5*) (Fig. 6). Six hours after CAPE spraying, we observed a strong induction of JA-responsive genes *PI-I* and *PI-II* and a slight induction of *TAS14*, *AREB1*, and *ERF5*. Surprisingly, the CAPE1 peptide induced strong expression of its own precursor gene *PR1b*, while SA-responsive genes *PR2* and *PR5x* were suppressed 3 h post-treatment. To understand the reason behind the differential behaviour of SA-responsive genes after CAPE1 treatment, we analysed the expression of SA biosynthesis genes. Expression of *ICS* and *PAL5* was slightly inhibited 3 h after CAPE1 treatment similar to SA-responsive genes *PR2* and *PR5x*. In contrast, CAPE treatment did not affect the expression of ABA-responsive genes (*TAS14* and *AREB1*) or ethylene-responsive gene (*ERF5*). Together, these results show that the application of CAPE1 peptide derived from PR1b results in a specific gene expression signature in tomato, enhancing the expression of JA-responsive genes and *PR1b*, while not affecting the expression of ABA-responsive and ethylene-responsive genes.

## Discussion

In this work we evaluated for the first time the xylem proteomes of resistant and susceptible tomato in response to *R. solanacearum*. One of the most overaccumulated proteins upon infection was the pathogenesis-related PR5x, which had been reported in the xylem sap of *F. oxysporum*-infected tomato (Rep *et al.*, 2002) and in the stem of *R. solanacearum*-infected LS-89 resistant tomato plants (Ishihara *et al.*, 2012). The role of PR5s in the interaction between plants and vascular pathogens is far from being understood. Although PR5x accumulates in both susceptible and resistant tomato lines upon fungal inoculation, it is accumulated to much higher levels in the latter, implying that it is involved in resistance (De Lamo *et al.*, 2018). In addition, a recent report showed that overexpressing the PR5 protein NP24 in tomato resulted in decreased susceptibility to *F. oxysporum* but enhanced susceptibility to *R. solanacearum* (Šimkovicová *et al.*, 2025). The subtilisin-like protease P69B, shown to be involved in tomato defence against *R. solanacearum* (Zhang *et al.*, 2024), was also identified as similarly accumulated in the xylem sap of Marmande and H7996, together with other PR proteins, as well as several Kunitz trypsin inhibitors (KTIs).

We also identified proteins with lower accumulation in the xylem of the susceptible variety Marmande, an effect that may be due to a combination of inhibition and cellular damage caused by the pathogen. Underaccumulated proteins included PR5s, peroxidases, glycoside hydrolases (GHs), and various proteases and peptidases (Fig. 1). Some PR5s and PR5-like proteins possess glucan binding and glucanase activity (Grenier *et al.*, 1999) or act as inhibitors of fungal xylanases (Fierens *et al.*, 2007), and have thus been proposed to have an anti-pathogenic activity. Peroxidases and GHs are involved in lignification and polysaccharide degradation, respectively, which take place in response to environmental stresses, such as pathogen attack (Minic and Jouanin, 2006; Miedes *et al.*, 2014). Overlignification and changes in the cell wall composition in response to *R. solanacearum* have been documented in the literature (Wydra and Beri, 2007; Ishihara *et al.*, 2012). Moreover, not only may GHs serve as simple cell wall ‘remodellers’, but the wall-derived damage-associated molecular patterns (DAMPs) released from their hydrolytic activity could also serve to amplify the signalling cascades that activate immunity (Bacete *et al.*, 2018). Reduced levels of peroxidases and GHs in the susceptible variety suggests that *R. solanacearum* might be actively preventing their anti-colonization function, although it could also result from cellular damage caused by the pathogen. In contrast, H7996 is able to reshape its cell wall structure, potentially reinforcing it and thus rendering the plant resistant.

Comparison of the xylem sap and the apoplastic fluid proteomes revealed PR1s as overaccumulated in response to *R. solanacearum* infection in the two compartments both in resistant and in susceptible plants. Thus, we decided to characterise them further (Fig. 1). However, the KTIs were the proteins most responsive to *R. solanacearum* infection (Supplementary Fig. S3). These inhibitors are specific for serine proteases but can also inhibit some cysteine and aspartic proteinases (Bonturi *et al.*, 2022). KTIs defend plants against herbivorous insects by inhibiting proteolytic enzymes from their guts (Bacete *et al.*, 2018), although they have also been reported as antimicrobials (Kim *et al.*, 2005). Hence, in the tomato–*R. solanacearum* context, KTIs might be induced to antagonize *R. solanacearum* colonization in the apoplast and xylem sap environments, probably by selective inhibition of its serine proteases.

Blasting PR1b against the tomato genome, we identified six PR1 proteins besides the well-described PR1a, b, c, and d. All these PR1 family proteins feature a conserved signal peptide, CAP domain, and CAPE peptide (Fig. 2A). The CAPE peptide, PxGNxxxxxPY, and the aspartic acid immediately preceding the CAPE peptide in the sequence are highly conserved in tomato, as observed in other species (Laugé and Goodwin, 2000; Ziegler *et al.*, 2000). In Arabidopsis, recent studies have proven that a cysteine protease cleaves PR1b to release the CAPE1 peptide (Chen *et al.*, 2023). However, in monocots like wheat, the cleaved amino acid is different (Sung *et al.*, 2021), revealing that different cleavage mechanisms may exist. All well-characterized plant species present some PR1s featuring an amino acid extension after the CAPE peptide, such as PR1c in tomato, which is highly conserved across solanaceous plants (Fig. 2A). Despite identifying new tomato PR1s in the genome, only the four previously described were detected in the apoplast or xylem (Figs 1C, D). PR1a, b, and c are the three family members most responsive to the bacterial pathogen *R. solanacearum*, being highly accumulated in both the apoplast and the xylem upon infection (Figs 3A, B). This may suggest redundant functions for these different proteins. Some PR1 proteins are known not to accumulate during infection (Cornelissen *et al.*, 1987). In this work, PR1d is only induced in the xylem, but not in the apoplast (Figs 3A, B), likely indicating some functional specificity in the vasculature.

Most PR1 proteins are thought to be secreted to the apoplastic space, due to their N-terminal signal peptide (Laugé and Goodwin, 2000; Ziegler *et al.*, 2000). PR1 of Arabidopsis is synthesized in the endoplasmic reticulum and then secreted into the apoplast through the late endosome/multivesicular body pathways (Pečenková *et al.*, 2017). However, PR1 versions with the cleavage motif mutated (YDPR to AAAA) are retained in the endoplasmic reticulum (Pečenková *et al.*, 2017, 2022). The tomato PR1b and its cleavage motif mutant fused to GFP showed the same localization as Arabidopsis PR1 (Supplementary Fig. S5), suggesting that the cleavage motif plays an important role in translocation of PR1 in different plant species.

Although PR1b is mostly localized in the apoplast, it has also been shown to locate in the vacuole (Dixon *et al.*, 1991; Funk *et al.*, 2002; Pečenková *et al.*, 2022). In the vacuole, the tracheary element vacuolar protein XCP1 was found to facilitate PR1b processing to release the CAPE1 peptide. Consequently, Arabidopsis plants deficient in *xcp1* showed an impaired response to pathogen *Pst DC3000* (Chen *et al.*, 2023). On the contrary, the XCP1 paralog XCP2 must have a different function, since Arabidopsis plants deficient in XCP2 displayed higher resistance to *R. solanacearum* compared with wild-type plants (Zhang *et al.*, 2014).

The exact function and mode of action of PR1s in defence is still unknown. Recent studies showed that the CAP domain has a conserved lipid binding function, contributing to anti-fungal defence, since PR1 can limit sterol-auxotrophic organisms through competition for sterol (Choudhary and Schneiter, 2012; Darwiche *et al.*, 2017; Gamir *et al.*, 2017). However, sterol is not necessary for growth of *R. solanacearum*. In accordance with this, the purified PR1b, PR1b^ΔCAPE^ and PR1b^KK^ did not affect growth of *R. solanacearum* (Supplementary Fig. S8). We tried to generate PR1b-deficient plants both in susceptible Marmande and resistant H7996 cultivars using CRISPR/Cas9. We obtained *pr1* mutant lines in Marmande (Supplementary Fig. S6), but we failed to obtain PR1b-deficient H7996 plants, which would have provided more information as this variety is highly resistant to *R. solanacearum*. Other teams reported their failure in obtaining *pr1b* knockout mutants in tomato (Li *et al.*, 2022), pinpointing a key role of this gene.

*Ralstonia solanacearum* pathogenicity assays using soil drenching inoculation showed that deletion of *PR1b* resulted in increased resistance (Fig. 4A). This is surprising considering that PR1b is supposed to facilitate plant defence. Interestingly, both the *pr1b*-deficient and overexpressing lines showed reduced *R. solanacearum* growth in the xylem (Fig. 4C), although no significant differences were observed in apoplastic growth (Fig. 4B). These contradictory results can be explained if in stems PR1b is involved in plant immunity regulation through different mechanisms than in roots. In addition, the loss of *PR1b* may activate other defence-related genes in the complex immune regulation network of tomato or up-regulate homologous genes in a compensatory effect as has been reported before (Gauffier *et al.*, 2016), enhancing the resistance of the mutant plants to *R. solanacearum*. Considering that PR1a and PR1d contain CAPE sequences similar to those of PR1b, deleting *PR1b* alone may lead to functional compensation by these closely related genes. Finally, alterations in PR1b levels (loss/overexpression) could in turn alter the levels of the protease involved in CAPE1 generation or its activation mechanisms, required for PR1/CAPE signalling.

The CAPE1 peptide derived from PR1b has been shown to act as an activator of immunity against bacterial and fungal pathogens (Chen *et al.*, 2014; Sung *et al.*, 2021; Li *et al.*, 2022). Here, we showed that spraying leaves with the CAPE1 peptide before inoculation with *R. solanacearum* limited bacterial multiplication (Fig. 5A). Addition of the peptide to *in vitro*-grown plants also limited *R. solanacearum* colonization of tomato plantlets both at the root and the hypocotyl (Fig. 5B–D). In contrast, the CAPE1 peptide did not affect growth of *R. solanacearum* in rich medium (Fig. 5E). Growing seedlings on plates supplemented with CAPE1 peptide clearly enhanced plant defence responses (Fig. 6), suggesting that recognition of CAPE1 peptide could occur both at the root and leaves.

To understand signalling triggered upon CAPE1 perception, we tested expression of different hormone-related plant defence genes. The results show that until 6 h after CAPE1 treatment, the peptide could highly induce JA-responsive genes *PI-I* and *PI-II* and PR1b (Fig. 6). On the contrary SA-responsive genes (*PR2* and *PR5x*) were not induced but slightly supressed 3 h after CAPE1 treatment and the expression of ABA-responsive genes (*TAS14* and *AREB1*) and ethylene-responsive gene (*ERF5*) was unaffected (Fig. 6). Since SA-responsive genes showed different regulation, we tested the expression of the upstream genes in the SA signalling pathway. Expression of SA-synthesis genes (*ICS* and *PAL5*) was inhibited by CAPE1 treatment, like *PR2* and *PR5x* (Fig. 6), indicating the existence of an unknown feedback loop in a PR1b-independent SA signalling pathway. Chen *et al.* (2014) showed that while CAPE1 peptide treatment strongly induced JA-responsive genes and PR1b, it suppressed the expression of other SA-responsive genes in the early stages after treatment. However, after 24 h of CAPE1 treatment, the peptide highly induced both SA-responsive and JA-responsive genes (Chen *et al.*, 2014). The JA and SA biosynthesis pathways have a very complex interrelation, often acting antagonistically (Robert-Seilaniantz *et al.*, 2011; Thaler *et al.*, 2012), although in some instances they may function synergistically, thereby redirecting the defence output (Verhage *et al.*, 2010). Considering our results, we speculate that CAPE1 may inhibit the multiplication of the hemibiotrophic pathogen *R. solanacearum* by inducing the SA-dependent pathway during the biotrophic phase and JA-triggered pathway during the necrotrophic phase. Given that both phases of bacterial infection can occur simultaneously in a natural infection, the activation of both pathways is not surprising. Alternatively, since phytocytokines like CAPE1 can act at a distance, such as in activation of systemic acquired resistance responses (Chen *et al.*, 2023), it is possible that the response in the tissue producing CAPE1 differs from that in distant tissues receiving the signal. This could lead to a differential response, where JA is activated in the PR1-producing tissue and SA is activated in the distant cells receiving the CAPE1 signal.

A recent study showed that the cysteine protease Xylem cysteine peptidase 1 (XCP1) could cleave PR1 to release the CAPE peptide in Arabidopsis (Chen *et al.*, 2023). The CAPE cleavage sites are conserved between Arabidopsis and *Solanaceae* plants, and future work will be crucial in determining whether tomato XCPs or other secreted proteases cleave PR1s to generate CAPEs. In addition to their roles in plant defence, PR1 proteins also exhibit virulence functions in certain pathogenic fungi and nematodes. For example, studies have shown that PR1 proteins are involved in the infection processes of fungal pathogens like *Fusarium oxysporum*, *Ustilago maydis*, and *Botrytis cinerea*, where they contribute to the manipulation of host immunity and enable the pathogen to establish a successful infection (Han *et al.*, 2023). Similarly, in nematode infections, PR1 proteins have been implicated in the interaction between plant hosts and root-knot nematodes, where they play a role in suppressing the plant’s immune response to facilitate nematode feeding and parasitism (Han *et al.*, 2023). These findings highlight the dual nature of PR1 proteins, which, while crucial for plant defence, can also be secreted by pathogens to enhance their virulence. Understanding the balance between these opposing roles will be important for developing strategies to harness the full potential of PR1 proteins to engineer resistance in plants.

## Supplementary data

The following supplementary data are available at *JXB* online.

Fig. S1. RNA levels of the reference gene used for RT-QPCRs.

Fig. S2. Proteomic changes in the xylem sap of susceptible and resistant tomato cultivars upon *R. solanacearum* infection.

Fig. S3. Identity of the xylem sap and apoplastic fluid proteomes upon *R. solanacearum* infection.

Fig. S4. Alignment and neighbour-joining protein similarity tree of tomato PR1 proteins.

Fig. S5. Subcellular localization of PR1b and PR1b^CNAD^ mutant.

Fig. S6. Generation of PR1b CRISPR/Cas9 deletion mutants in Marmande tomato plants.

Fig. S7. Generation of Marmande tomato plants overexpressing PR1b and PR1b^ΔCAPE^.

Fig. S8. Purification of recombinant PR1b variants and analysis of their effect on *R. solanacearum* growth *in vitro*.

Table S1. Databases used for the construction of PR1-like gene phylogeny in different species.

Table S2. Gene and protein identifiers for tomato PR1s.

Table S3. List of primers used in this work.

Table S4. Guide sequences (sgRNA) used for the generation of tomato PR1b mutants by CRISPR/Cas9 and primers for verifying.

Dataset S1. Proteomics processed data.

eraf145_suppl_Supplementary_Tables_S1-S4_Figures_S1-S8

eraf145_suppl_Supplementary_Datasets

## Data Availability

The mass spectrometry proteomics data for the on-bead digestions have been deposited to the ProteomeXchange Consortium via the PRIDE (Vizcaíno *et al.*, 2016) partner repository (https://www.ebi.ac.uk/pride/archive/) with the dataset identifier PXD058990.
