# Supplementary material for: The CAPE1 peptide confers resistance against bacterial wilt in tomato
Source: J Exp Bot. 2025 Apr 3;76(15):4340–58. doi: 10.1093/jxb/eraf145 (PMC12485366; doi:10.1093/jxb/eraf145)
Supplement: eraf145_suppl_Supplementary_Tables_S1-S4_Figures_S1-S8 [file eraf145_suppl_supplementary_tables_s1-s4_figures_s1-s8.pdf]

SUPPLEMENTARY INFORMATION FOR:

The CAPE peptide confers resistance against bacterial wilt in tomato

Running title: Tomato xylem sap proteome analysis and PR1b role in resistance to *Ralstonia solanacearum*

Weiqi Zhang[1], Marc Planas-Marquès[1,2], Moyan Liang[1,2], Qingshan Zhang[1], Annemarie Vermeulen[3], Farnusch Kaschani[4], Markus Kaiser[5], Frank L. W. Takken[3], Nuria S. Coll[1,6*], Marc Valls[1,2*]

[1] Centre for Research in Agricultural Genomics (CRAG), CSIC-IRTA-UAB-UB, Campus UAB, 08193 Bellaterra, Spain.

[2] Department of Genetics, Microbiology and Statistics, Universitat de Barcelona, 08028 Barcelona, Catalonia, Spain.

[3] Molecular Plant Pathology, Faculty of Science, Swammerdam Institute for Life Sciences, University of Amsterdam, Amsterdam, Netherlands.

[4] Analytics Core Facility Essen (ACE), Chemical Biology, Faculty of Biology, Universität Duisburg-Essen, ZMB, 45117 Essen, Germany

[5] Chemical Biology, Faculty of Biology, Universität Duisburg-Essen, ZMB, 45117 Essen, Germany

[6] Consejo Superior de Investigaciones Científicas (CSIC), 08001 Barcelona, Spain.

[*] Authors for correspondence:

Nuria S. Coll. Centre for Research in Agricultural Genomics. 08193 Bellaterra. Spain

e-mail: nuria.sanchez-coll@cragenomica.es

Marc Valls. Universitat de Barcelona, 08028 Barcelona, Catalonia, Spain

e-mail: marcvalls@ub.edu

Figure S1

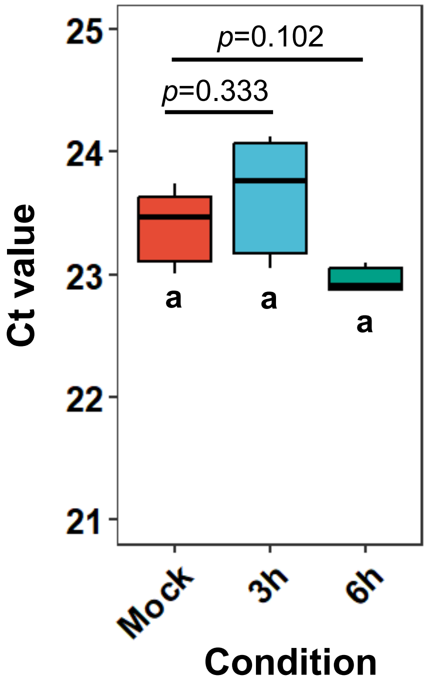

Figure S1. RNA levels of the reference gene used for RT-QPCRs. RNAs extracted from the aerial part of four-week-old tomato plants sprayed with water or 3 or 6 hours after treatment with the CAPE peptide were retro-transcribed and subjected to quantitative PCR. Threshold cycle (Ct) values for the reference actin (*SlACT2)* gene are shown. Student's t-test p values for the differences between treated CAPE peptide at different hours and mock and analysis of variance (ANOVA) results amongst all conditions are shown. Same letters indicate no significant difference in the ANOVA test.

Figure S2

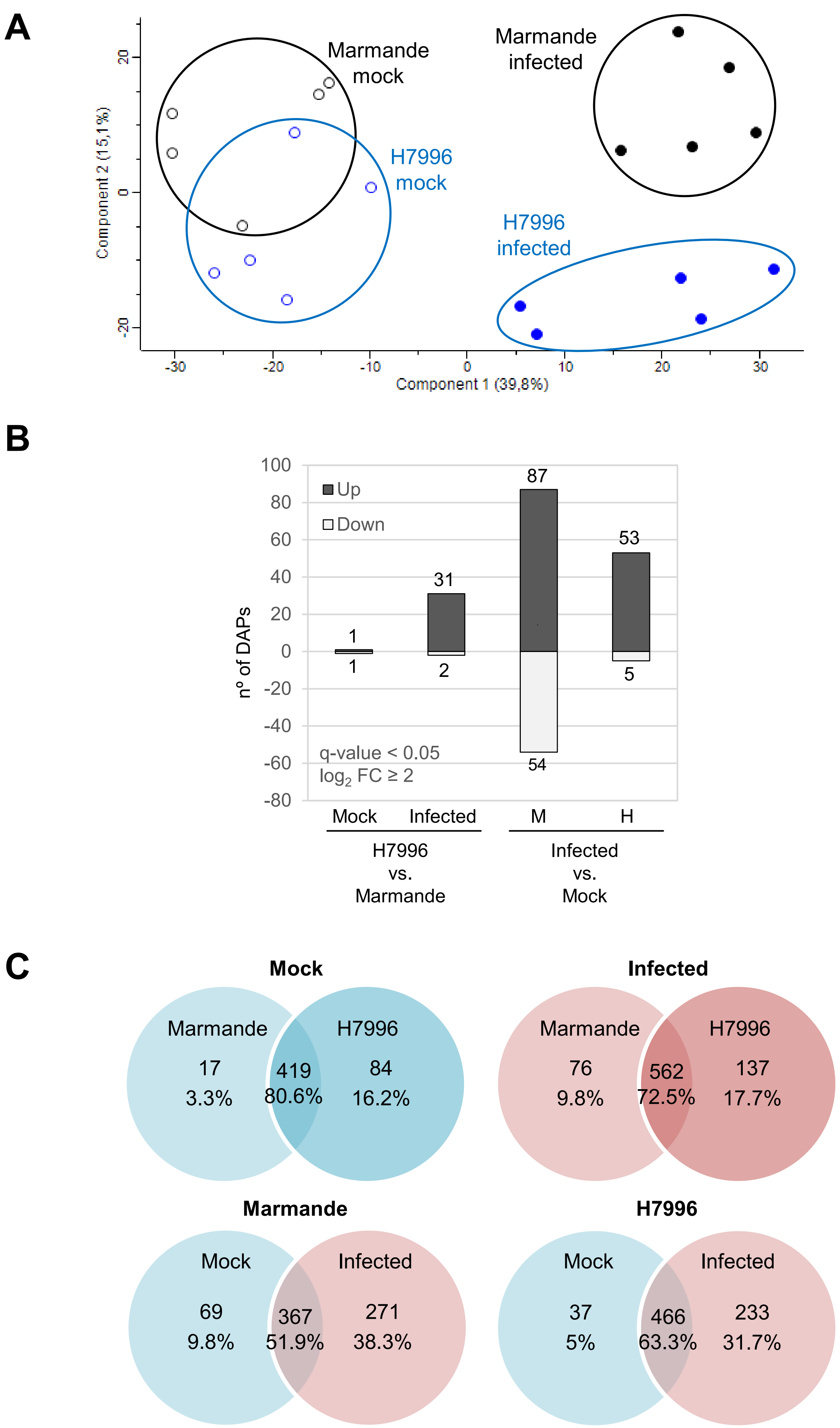

A
Marmande
mock
Marmande
infected
H7996
mock
H7996
infected
Component 2 (15,1%)
Component 1 (39,8%)
20
0
-20
-30
-20
-10
0
10
20
30
B
Up
Down
87
53
31
1
1
2
5
54
100
80
60
40
20
0
-20
-40
-60
-80
nº of DAPs
q-value < 0.05
log2 FC ≥ 2
Mock
Infected
M
H
H7996
vs.
Marmande
Infected
vs.
Mock
C
Mock
Marmande
H7996
17
3.3%
419
80.6%
84
16.2%
Infected
Marmande
H7996
76
9.8%
562
72.5%
137
17.7%
Marmande
Mock
Infected
69
9.8%
367
51.9%
271
38.3%
H7996
Mock
Infected
37
5%
466
63.3%
233
31.7%

Figure S2. Proteomic changes in the xylem sap of susceptible and resistant tomato cultivars upon *R. solanacearum* infection. Xylem sap protein profile of the Marmande (M) and H7996 (H) tomato cultivars inoculated with water (mock) or *R. solanacearum*. Xylem saps were collected 3 days post-inoculation, several replicates were pooled and the samples were equally concentrated. **(A)** Principal component analysis (PCA) of the xylem sap proteome. **(B)** Differentially accumulated proteins (DAPs) by treatment and variety. 'Up' and 'Down' indicate over- and underaccumulated proteins, respectively. Only DAPs with an FDR q-value < 0.05 and $\log_2$ FC ≥ 2 are represented. DAPs were detected after imputing the missing protein abundance values. **(C)** Venn-diagrams showing the total number of the xylem sap proteins identified in Marmande and H7996 upon infection. Non-imputed proteomic data was considered, filtered by valid values (non-missed detections, see *Methods* section) and all majority proteins within each protein group were accounted in the total number of proteins.

Figure S3

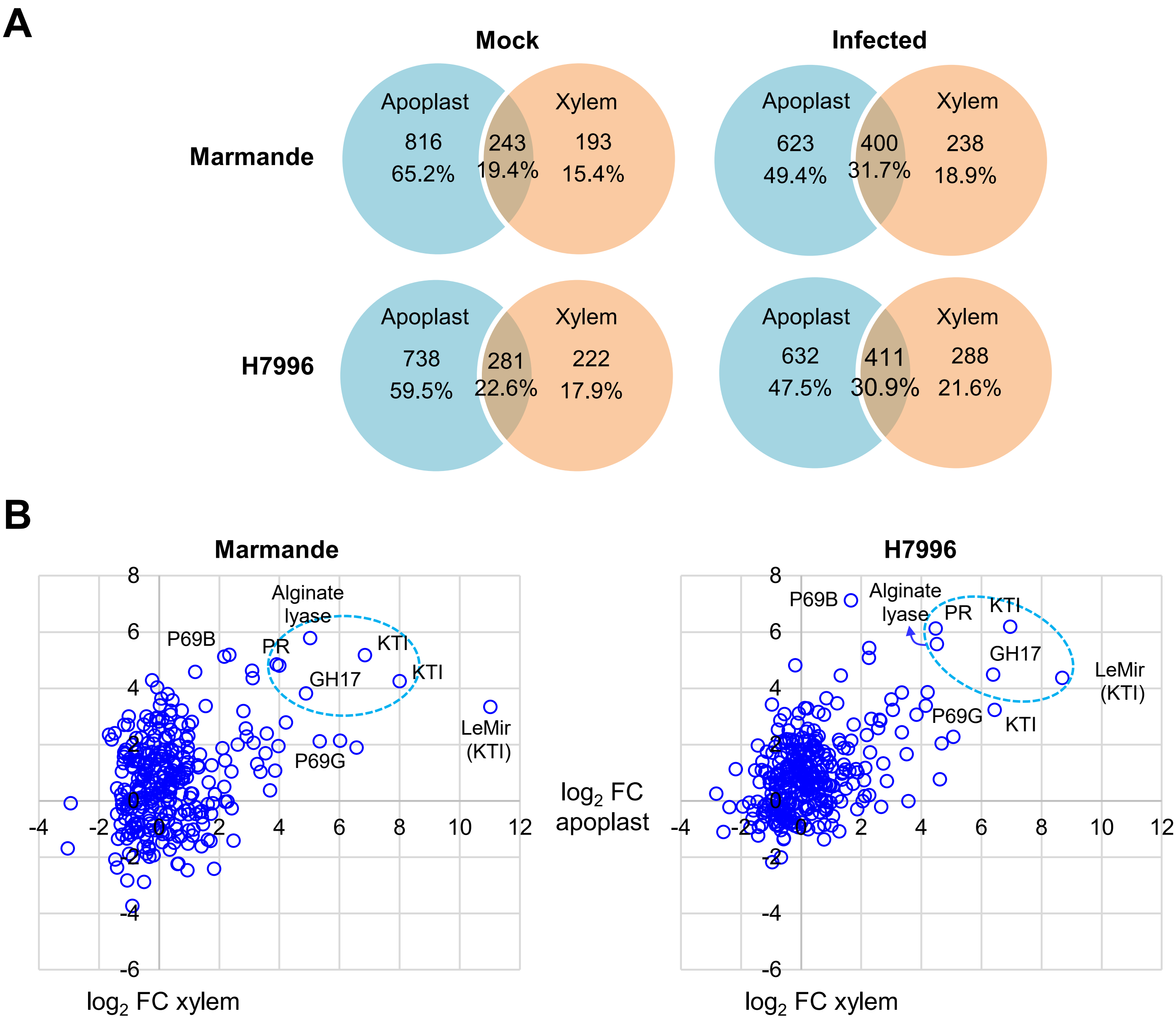

Figure S3. Identity of the xylem sap and apoplastic fluid proteomes upon *R. solanacearum* infection. (A) Venn-diagrams representing the number of overlapping proteins between the xylem and apoplastic proteomes of each tomato variety (Marmande and H7996) in basal conditions (mock) and upon infection. Non-imputed proteomic data was considered, filtered by valid values and all majority proteins within each protein group were accounted in the total number of proteins. **(B)** Dispersion plots showing the $\log_2$ fold change (FC) of the overlapping proteins between the xylem and apoplastic proteomes upon infection in the two varieties. Log_2 FC values were retrieved after imputing missing values. Proteins with $\log_2$ FC ≥ 4 in both proteomes are highlighted. PR, pathogenesis-related; GH, glycoside hydrolase; KTI, Kunitz trypsin inhibitor.

Figure S4

A

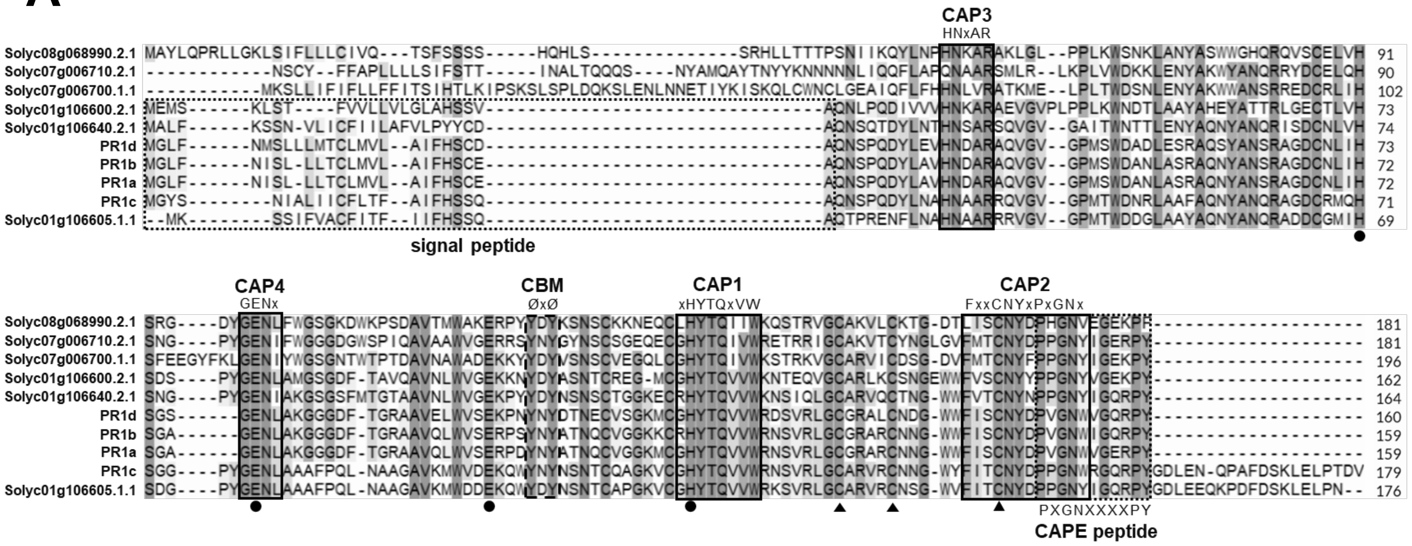

B

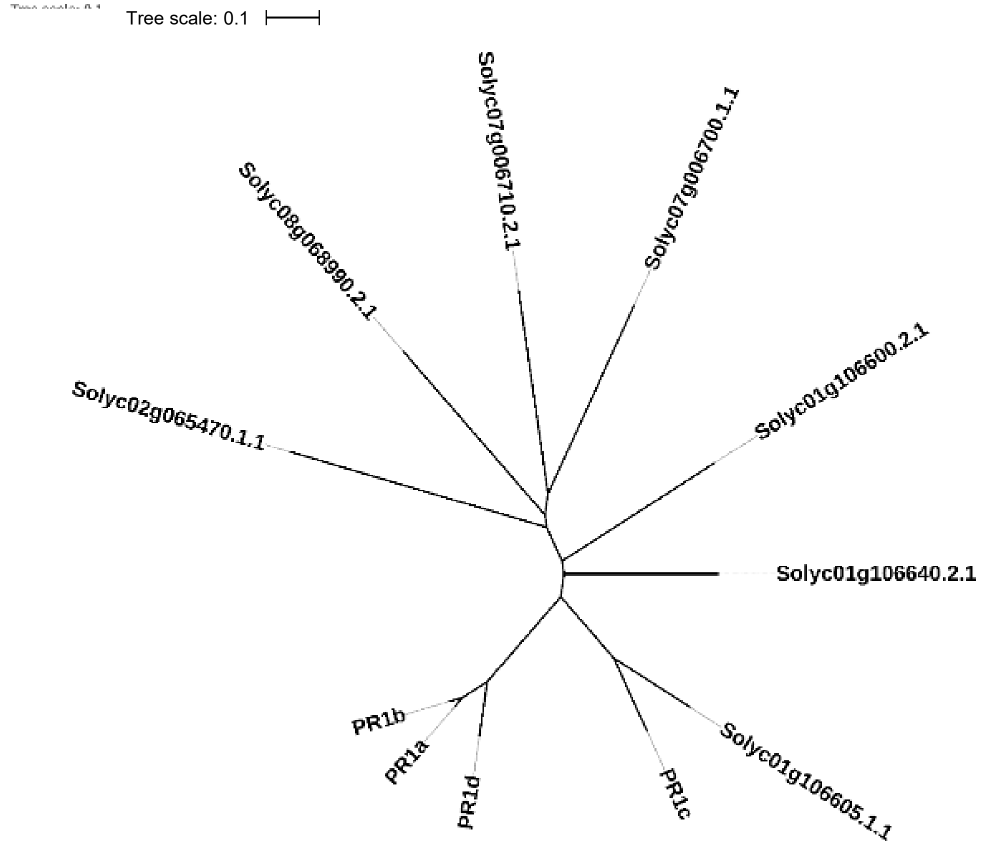

Figure S4. Alignment and neighbour-joining protein similarity tree of tomato PR1 proteins. **(A)** Sequence alignment of PR1 family members from tomato plants. The CAP1–4 signature motifs are boxed in black. The dashed black line indicates the conserved aromatic amino acids (ØXØ) that define the caveolin-binding motif (CBM). The solid black line indicates the signal peptide and conserved CAPE peptide. Black circles indicate the highly conserved histidine and glutamic acid residues that constitute the conserved tetrad residues. Arrowheads indicate the three cysteines that are highly conserved in all proteins. **(B)** Neighbour-joining protein similarity tree of tomato PR1 proteins generated from a ClustalO alignment. The most highly related non-PR1 Solyc02g065470.1.1 was added as an outgroup. Scale bar indicates 0.1 amino acid changes per site.

Figure S5

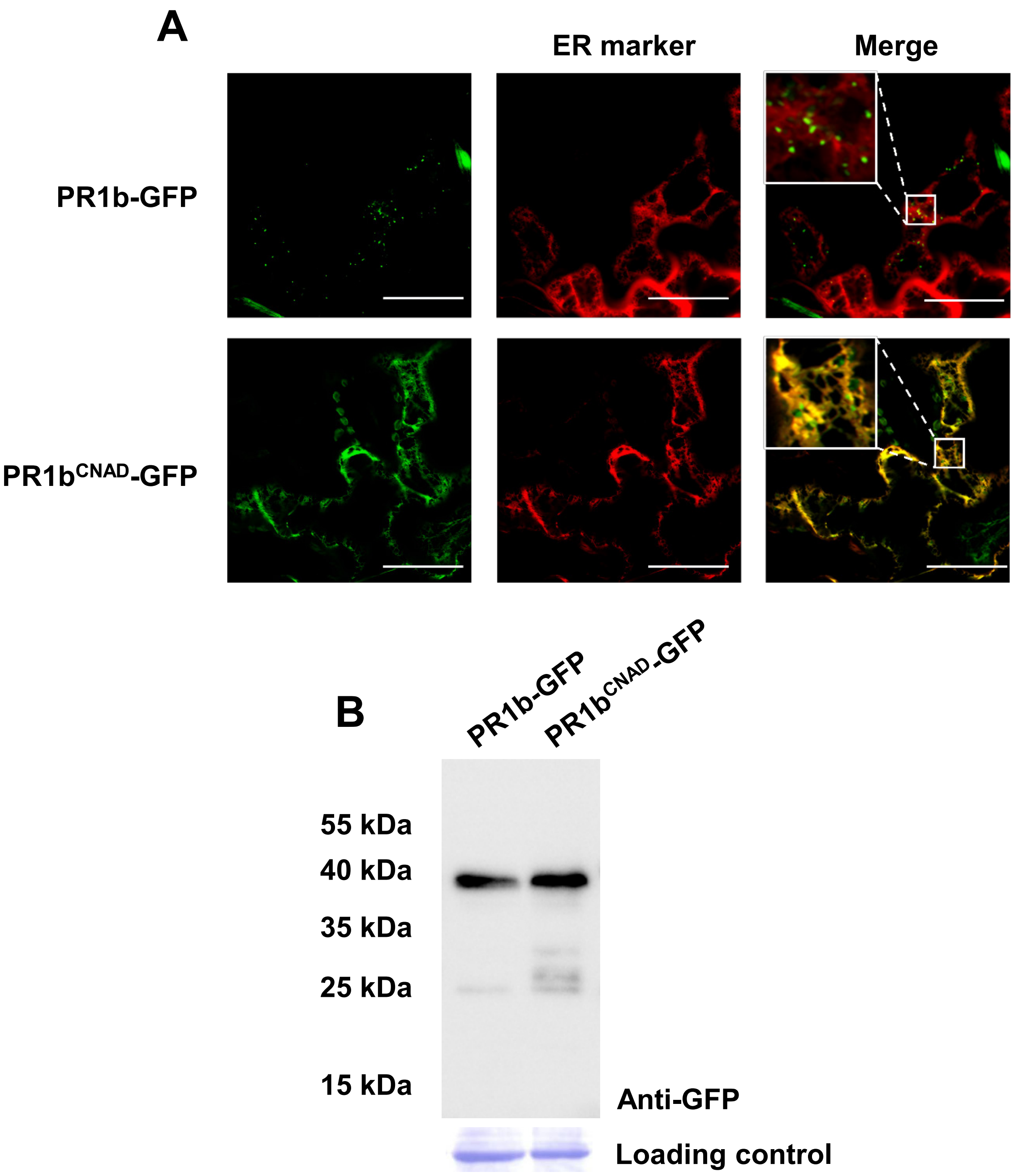

Figure S5 Subcellular localization of PR1b and PR1b^{CNAD} mutant. A) *PR1b* and *PR1b^{CNAD}* fused to *GFP* were transiently expressed in *Nicotiana benthamiana* using Agrobacterium-mediated transformation. Confocal microscopy images of *N. benthamiana* leaves co-expressing *PR1b-GFP* or *PR1b^{CNAD}–GFP* together with the endoplasmic reticulum marker ER-rk CD3-959. Scale bar: 20 µm. Insets depict a magnified view of the signal in the solid squares. **B)** Western blotting of total proteins extracted from the same leaves observed in A) and Coomassie blue staining of the membranes to show total protein abundance

Figure S6

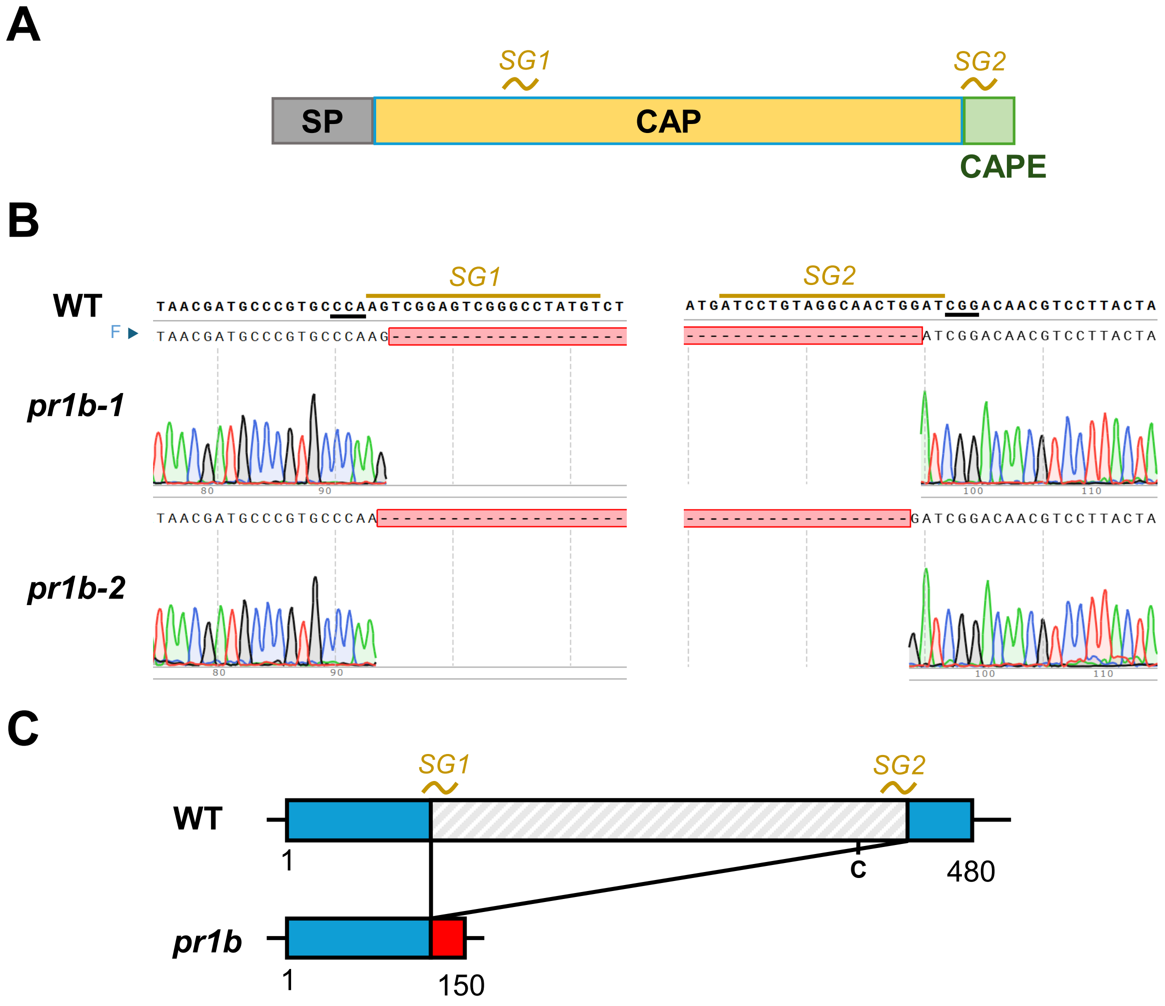

Figure S6. Generation of PR1b CRISPR/Cas9 deletion mutants in Marmande tomato plants. (A) Schematic depiction of the location of the used sgRNAs in the PR1b protein sequence **(B)** Sequence results of PR1b mutants. Arrowhead indicates the position of the genotyping forward primers. The protospacer-adjacent motif (PAM) sequence is underlined and the sgRNA sequences indicated. Arrows and dashed lines indicate the putative double-strand break site used by Cas9. **(C)** Schematic representation showing the 112 bp deletion obtained by CRISPR/Cas9 in the PR1b gene. The mutation results in a late stop codon, which is then translated into a truncated protein variant. C indicates the cysteine residues from the putative active site. We detected shift deletions in the PR1 gene in 2 out of 63 plants analyzed (*pr1b*-1 and *pr1b*-2). Cas9 was segregated out and mutations brought to homozygosity in subsequent generations.

Figure S7

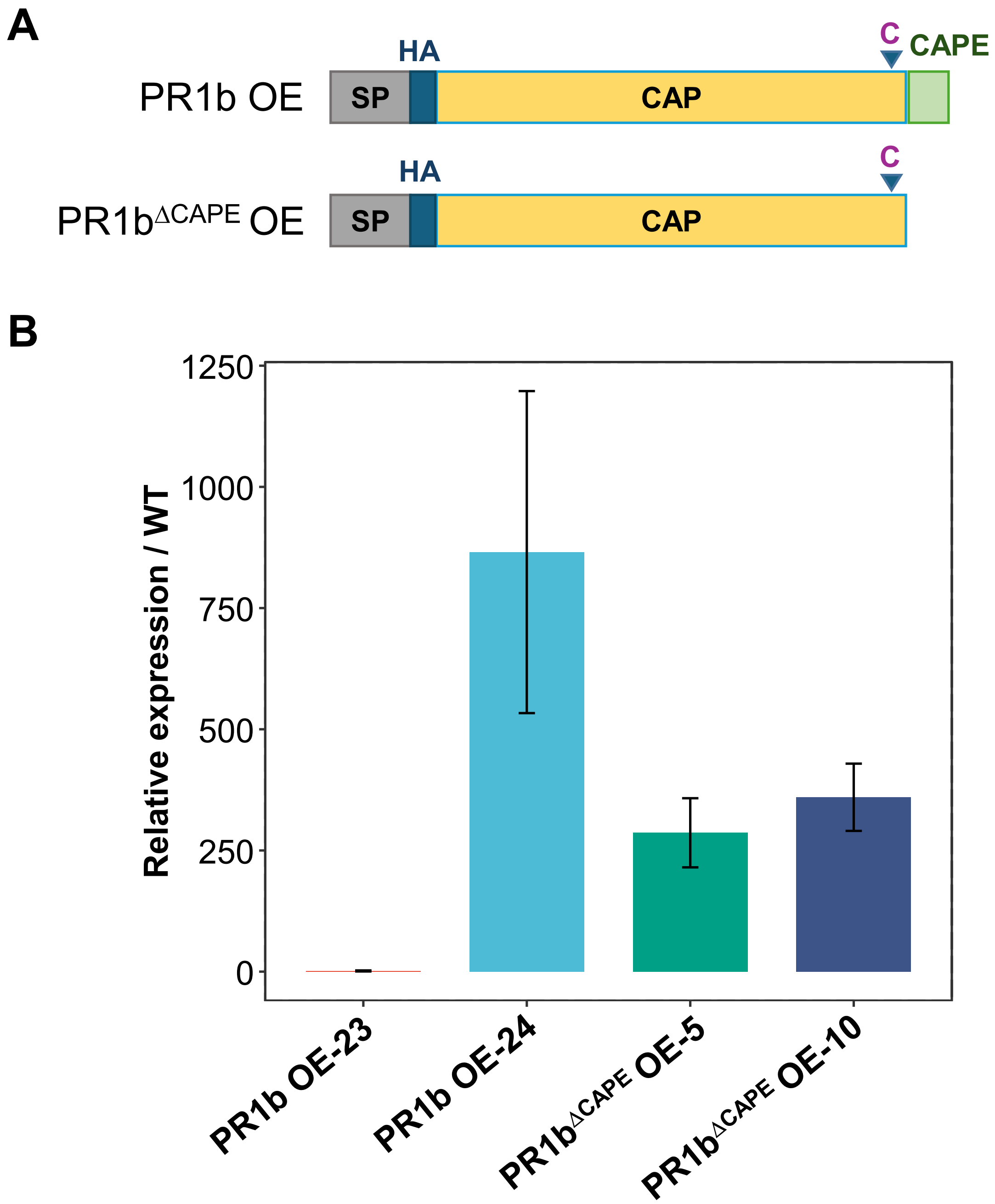

Figure S7. Generation of Marmande tomato plants overexpressing PR1b and PR1b$^{\Delta CAPE}$. (A) Schematic representation of the PR1 versions used in the overexpression lines. SP, signal peptide; HA, hemagglutinin tag; CAP, Cysteine-rich secretory proteins, Antigen 5, and Pathogenesis-related 1 proteins domain; CAPE, CAP-derived peptide. **(B)** PR1b gene expression levels comparing overexpression lines with wild type by qRT-PCR. The *Actin* gene (*SlAct2*) was used as endogenous reference. Three biological replicates were performed.

Figure S8

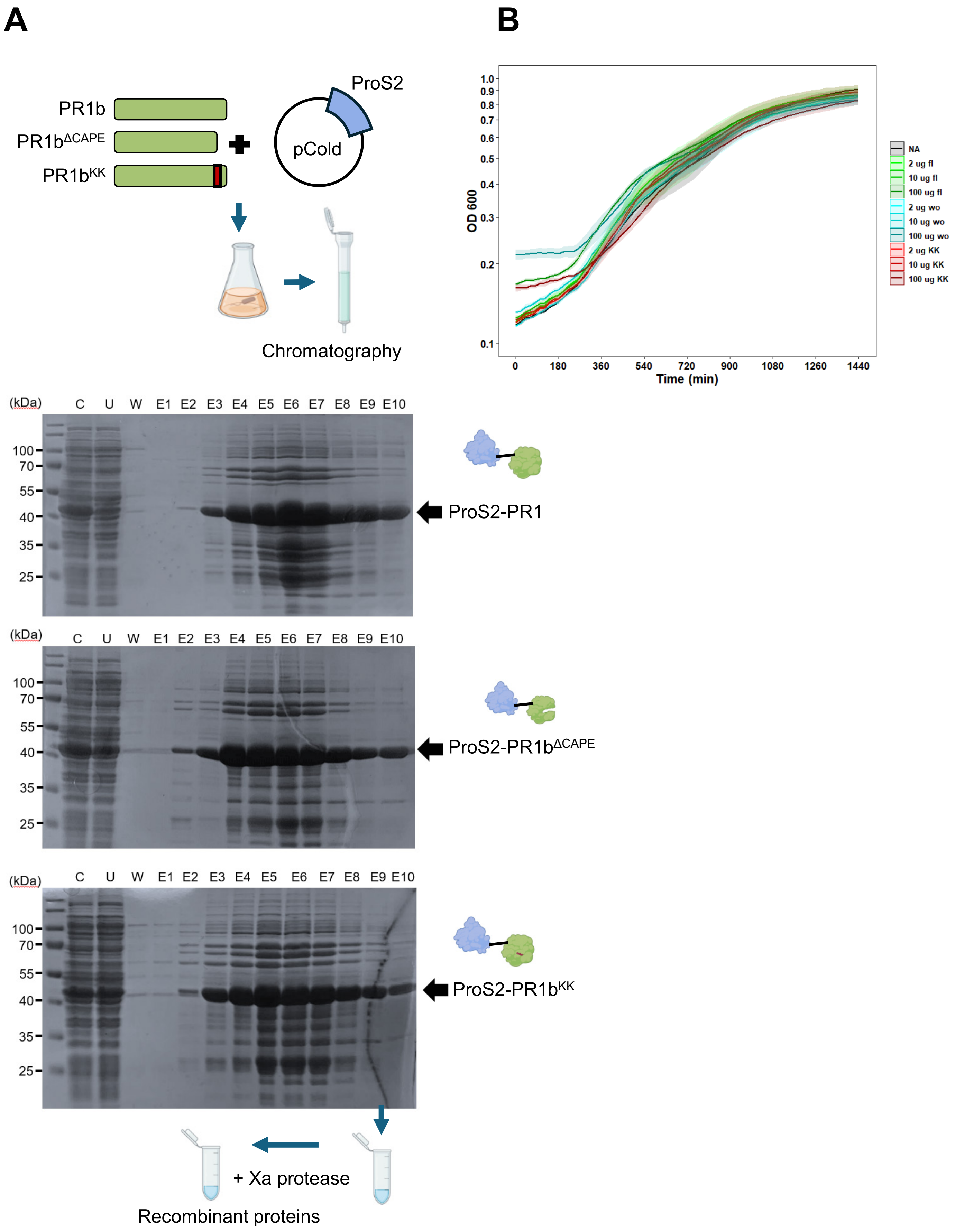
A
B
PR1b
PR1b$^{\Delta CAPE}$
PR1b^{KK}
ProS2
pCold
Chromatography
OD 600
Time (min)
0 180 360 540 720 900 1080 1260 1440
0.1 0.2 0.3 0.4 0.5 0.6 0.7 0.8 0.9 1.0
NA
2 ug fl
10 ug fl
100 ug fl
2 ug wo
10 ug wo
100 ug wo
2 ug KK
10 ug KK
100 ug KK
(kDa)
C U W E1 E2 E3 E4 E5 E6 E7 E8 E9 E10
100 70 55 40 35 25
ProS2-PR1
ProS2-PR1b$^{\Delta CAPE}$
ProS2-PR1b^{KK}
+ Xa protease
Recombinant proteins

Figure S8. Purification of recombinant PR1b variants and effect on *R. solanacearum* growth *in vitro*. (A) Expression of recombinant PR1b variants in *Escherichia coli* was induced by IPTG. Protein extracts were subjected to metal chelate affinity chromatography. Fractions of each purification step were loaded onto an SDS-PAGE and proteins were stained using Coomassie Brilliant Blue (CBB). C: Crude; U: Unbound; W: Wash, E: Elution fraction. Arrows indicate target proteins. **(B)** Effect of purified PR1 variants on *R. solanacearum* growth *in vitro*. *R. solanacearum* at 10^8 CFU/mL was incubated in rich B medium with different concentrations of recombinant PR1b and derivatives. Bacterial growth was recorded over time. PR1bfl: PR1b full length, green. PR1bwo: PR1b without CAPE peptide, blue. PR1bKK: PR1b CNKK, red. The light area means standard error.

Table S1. Databases used for the construction of PR1-like gene phylogeny in different species

Organism	Database
Solanum lycopersicum	Tomato Genome proteins (ITAG release 4.0)
Solanum tuberosum	Potato PGSC DM v3.4 Protein sequences
Capsicum annuum	Pepper cv. CM334 Genome protein sequences (release 1.55)
Nicotiana tabacum	Tobacco Nitab v4.5 proteins Edwards 2017 (draft genome)
Arabidopsis thaliana	Arabidopsis TAIR10 protein
Triticum aestivum	NCBI: AEH25616.1

Table S2. Gene and protein identifiers for tomato PR1s.

Gene Accession (NCBI)	UniprotKB	SGN	PR1 name
NP_001234314	P04284	Solyc00g174340.1.1	PR1b
NP_001234523	Q04108	Solyc09g007010.1.1	PR1a
NP_001307969	O24026	Solyc09g007020.1.1	PR1d
NP_001234358	B2LW68	Solyc01g106620.2.1	PR1c
NP_001234128	K4B2M8	Solyc01g106605.1.1	
XP_004230669	K4B2N1	Solyc01g106640.2.1	
XP_004230668	K4B2M7	Solyc01g106600.2.1	P4-like
XP_004242676	K4CBE2	Solyc07g006710.2.1	PR1-like
XP_004242675	K4CBE1	Solyc07g006700.1.1	PR1-like
XP_004245428	A0A3Q7HSC4	Solyc08g068990.2.1	

Table S3. List of primers used in this work.

Primer name	Sequence (5' to 3')
PI-I F	CCGGTTCCTTCACTCTTTACAAC
PI-I R	GCCCATCAATTTCTTTTCGTGC
PI-II F	CACAGGGTACAAGGGTTGCTAT
PI-II R	GAAGATGGACAAGTCTAGAGTCACA
PAL5 F	ACCTATCTCGTGGCGCTTTG
PAL5 R	TCTTTTTGGCTACTTGGCTTACAG
ICS F	TGATGAAACTTGAGGCACCTCTT
ICS R	GTCTACTAAATACGATGCGGCAG
ERF5 F	ACATCGCAAGCTTCTTTAATGCT
ERF5 R	TCCGTTCTCTCAAGTTACTCTGT
PR1b F	GGTGTCCGAGAGGCCAAG
PR1b R	TGCCCGACCACAACCTAGT
PR2 F	TCGGAAAGTGGTTGGCCTTC
PR2 R	GTTCCTGCCCCTCCTTTCAC
PR5x F	ACAAAGGTGCCCTAATGCGTA
PR5x R	TGTGGCATCAATTATGGGCAA
TAS14 F	TCGTCGATCCGACAGCTCTA
TAS14 R	ACCTTCATGTTGTCCAGGCA
AREB1 F	GTTGGTGTTCCAGGTGGTCA
AREB1 R	CCACCACCATGCTCCTTTGA
PR1a F GG	AACAGGTCTCAAACAATGGGGTTGTTCAACATCTCAT

PR1a R GG	AACAGGTCTCTAGCCATAAGGACGTTCTCCAACCCAGTT
PR1b R GG	AACAGGTCTCTAGCCGTAAGGACGTTGTCCGATCCAGTT
PR1c F GG	AACAGGTCTCAAACAATGGGATACTCCAATATTGCTT
PR1c R GG	AACAGGTCTCTAGCCGACATCAGTTGGAAGTTCCAACTT
PR1d F GG	AACAGGTCTCAAACAATGGGGTTGTTTAACATGTCAT
PR1d R GG	AACAGGTCTCTAGCCGTAAGGACGTTGTCCGACCCAATT
PR1a CNAD F	GGTGGTTCATTTCTTGCAACGCCGATCCTGTAGGCAACTGGGT
PR1a CNAD R	ACCCAGTTGCCTACAGGATCGGCGTTGCAAGAAATGAACCACC
PR1b CNAD F	GGTGGTTCATTTCTTGCAACGCCGATCCTGTAGGCAACTGGAT
PR1b CNAD R	ATCCAGTTGCCTACAGGATCGGCGTTGCAAGAAATGAACCACC
PR1c CNAD F	GGTACTTTATAACTTGTAATGCCGATCCACCAGGTAACTGGAG
PR1c CNAD R	CTCCAGTTACCTGGTGGATCGGCATTACAAGTTATAAAGTACC
PR1d CNAD F	GGTGGTTTATTTCTTGCAACGCCGATCCTGTAGGCAATTGGGT
PR1d CNAD R	ACCCAATTGCCTACAGGATCGGCGTTGCAAGAAATAAACCACC
35S NotI	ATGCGGCCGCTGAGACTTTTCAACAAAG
PR1b ol R	GAAGCGTAATCTGGAACATCGTATGGGTAATTTTGGGCCTCACAAGA
PR1b ol F	ATTACCCATACGATGTTCCAGATTACGCTTCACCCCAAGACTATCTT
PR1b fl NotI	ATGCGGCCGCTTAGTAAGGACGTTGTCCGAT
PR1b wo NotI	ATGCGGCCGCTTAATAGTTGCAAGAAATGAA
PR1b XhoI HA	ATCTCGAGATACCCATACGATGTTCCAGATTACGCTGCCCAAAATTCACCCCAAGA
PR1b HindIII HA	GTAAGCTTTTAGTAAGGACGTTGTCCGA
PR1b wo HindIII HA	GTAAGCTTTTAATCATAGTTGCAAGAAATGA
PR1b CNKK F	GGTGGTTCATTTCTTGCAACAAGAAGCCTGTAGGCAACTGGGTTGG
PR1b CNKK R	CCAACCCAGTTGCCTACAGGCTTCTTGTTGCAAGAAATGAACCACC

Table S4. Guide sequences (sgRNA) used for the generation of tomato PR1b mutants by CRISPR/Cas9 and primers for verifying. The protospacer adjacent motif (PAM) sequence recognized by Cas9 is underlined.

Strategy	Sequence (5’ to 3’)
sgRNA for PR1b mutant	U3-SG1: ACATAGGCCCGACTCCGACTTGG
	U6-SG2: ATCCTGTAGGCAACTGGATCGG
Detecting PR1b mutant	F: ATGGGGTTGTTCAACATCTC
	R: TTAGCAACATCAAAAGGGAA
Detecting PR1a mutant	F: ATGGGGTTGTTCAACATCTC
	R: CATGAACATATGGTACGTGG
Detecting Cas9	F: TCCCTTACTACGTGGGACCTC
	R: ATCTGCCTGGTTTCCACAG
